# Preparing for Potential Health and Safety Risks at the Olympic Games: Scoping Review

**DOI:** 10.2196/66829

**Published:** 2025-09-03

**Authors:** Shaotong Ren, Tiantian Li, Yongzhong Zhang, Song Bai, Zichen Zhou, Shengxin Li

**Affiliations:** 1 School of Disaster and Emergency Medicine, Tianjin University, 92 Weijin Road Nankai District, Tianjin, 300072, China, 86 15902222185; 2 College of Public Health, Zhengzhou University, Zhengzhou, China; 3 School of Management, Beijing Sport University, Beijing, China

**Keywords:** Olympic Games, illness, injury, terrorism, surveillance, PRISMA

## Abstract

**Background:**

The Olympic Games are an example of a mass gathering that involves a complex and large crowd composition, with a large number of illnesses and injuries occurring at previous Olympic Games, and the Olympic Games also becoming a target for terrorist attacks.

**Objective:**

With the help of mass-gathering medicine as a guide, this study aims to critically summarize and analyze the state of illness, injury, and terrorism during the Olympic Games in order to reduce the incidence of illnesses and injuries in crowds and to offer lessons for the organization of major international sporting events such as the Olympics.

**Methods:**

The procedure for this scoping review followed the 5-step methodological framework of Arksey and O’Malley. We searched electronic databases such as PubMed, Web of Science, and Scopus. We extracted, summarized, and categorized general information on each study, game characteristics, illness and injury profiles, terrorism characteristics, preventive measures, and surveillance paradigms.

**Results:**

We conducted a database search and retrieved a total of 9587 studies on 2 occasions. After removing duplicates and screening, we included 120 studies. Only 12 studies on the Summer, Winter, and Paralympic Games published before 2000, and 108 studies from 2000 onward, comprise the 120 studies, marking an unprecedented number of studies in this field of research, particularly in recent times. Of the 120 studies, 80 were illness-related, 81 were injury-related, and 2 were terrorism-related. Nine studies explicitly assessed body parts, including shoulders, feet, and dentistry; 26 studies specifically investigated certain illnesses and injuries, such as COVID-19 disease, heat-related illnesses, and concussions. Of the 120 research studies, 18 specifically analyzed sports such as gymnastics and weight lifting, with 11 studies focusing especially on COVID-19 disease. The most studied games were the Tokyo 2021 Olympic or Paralympic Games, the London 2012 Olympic or Paralympic Games, and the Rio 2016 Olympic or Paralympic Games. The system of injury and illness surveillance in the Olympic Games goes through 3 stages of development: the first trial of information technology, the construction of networks, and the enhancement of intelligence.

**Conclusions:**

A critical summary of studies of illness, injury, and terrorist attacks at previous Olympic Games is important for injury and terrorism prevention at major sporting events such as the Olympic Games. Surveillance methods require improvements in surveillance technology, data sharing, and privacy protection.

## Introduction

According to the World Health Organization definition, a mass gathering is “any organized or spontaneous event that attracts a sufficient number of people to strain the planning and response resources of the community, city, or country hosting the event.” [1]. Mass gatherings include World Youth Day, the Olympic Games, and political rallies [2]. The Olympic Games are the world’s largest and most influential sporting event, with a particularly large and complex population that involves many stakeholders. Since the XXVI Olympic Games in Atlanta in 1996, hundreds of thousands of athletes, volunteers, spectators, and other stakeholders have consistently participated in each Olympic Game. Such large-scale crowd gatherings can pose significant public health concerns for the well-being and safety of participants, host country inhabitants, and even the worldwide human population [1].

Previous studies of major health and safety issues at the Olympic Games have focused sporadically on illness, injury, and terrorist attacks [3,4]. The studies focused on groups of tens of thousands of people, such as athletes and Olympic personnel, and found that the most common illnesses were respiratory, gastrointestinal, and heat-related illnesses [5-7]. Meanwhile, numerous studies have explored the sites, types, mechanisms, and incidence of injuries in athletes from previous Olympic Games, as well as the competitions with the highest risk. In recent years, traditional high-risk and emerging uncertain infectious illnesses such as the global epidemic and variation of COVID-19 disease, the active global cholera epidemic [8], and the unknown future of mankind’s Illness X [9] have posed a significant threat to major international sporting events such as the Olympic Games. Furthermore, terrorism concerns have historically drawn attention to previous Olympic Games as a security issue. Simultaneously, bioterrorism has the characteristics of easy transport, low cost, hidden dissemination, and wide influence [10], and it takes advantage of the global influence of the Olympic Games, which makes it very easy to become a potential high-risk threat.

Protecting the health of athletes is an important task for the International Olympic Committee (IOC) [11]. The IOC and host nations, among other stakeholders, implement several strategies to mitigate and manage infections, injuries, and acts of terrorism. Regarding illness and injury prevention, host countries implement immunization programs, enhance publicity and education, prioritize personal hygiene, devote attention to medical personnel training, and modify the competition rules [12-14]. Furthermore, surveillance systems such as the Web-based Injury and Illness Surveillance System and Event-based Surveillance are adopted for monitoring [15,16]. Regarding counterterrorism responses, actions encompass organizing drills, providing funding, and strengthening security. Examples include the deployment of a disaster medical assistance team and a highly sophisticated chemical-biological incident response unit during the 1997 Atlanta Olympic Games. [17,18]. Meanwhile, mass-gathering medicine is a specialized discipline [19] that focuses on the provision of medical care and health protection during large crowd gatherings, providing some guidelines for the prevention of illnesses, injuries, and terrorism arising from the Olympic Games.

Extensive studies have demonstrated the prevalence of illnesses and injuries during the Olympic Games, along with multiple instances of terrorism, posing a significant risk to the physical and emotional well-being and security of athletes and individuals participating in the Games. However, systematic studies on the prevention and control of illness, injury, and terrorism in the Olympic Games are rare, and analytical studies of the evidence on the factors contributing to these health and safety impacts are still very sporadic. To fill this gap, the objective of this scoping review is to provide an overview and critical summary of the available evidence on risk factors, preventive measures, and surveillance approaches to illness, injury, and terrorism at the Olympic Games, with a view to providing counterstrategies to help major national and international sporting events in the future reduce health and safety risks.

## Methods

### Design and Search Strategy

The procedure for the scoping review followed Arksey and O’Malley’s 5-step methodological framework, including (1) identification of the study problem, (2) identification of relevant studies, (3) study selection, (4) extraction and graphing of data, and (5) collation, summarization, and reporting of results [20]. Results were presented in accordance with the PRISMA-ScR (Preferred Reporting Items for Systematic Reviews and Meta-Analyses extension for Scoping Reviews) recommendations [21].

On April 1, 2024, we conducted a systematic search of electronic databases (PubMed, Web of Science, and Scopus). The search strategy was divided into two parts: (1) the sports event, such as “Olympic,” “Paralympic,” or “Winter Olympic Games”; and (2) terms that covered topics such as “injury,” “illness,” “infectious diseases,” or “terrorism” (see Multimedia Appendix 1 for the detailed search strategy [22-87]).

### Inclusion and Exclusion Criteria

The inclusion and exclusion criteria applied in this study are detailed in Textbox 1.

Textbox 1. **Inclusion and exclusion criteria.**

Inclusion criteria

- Studies explicitly addressing the Olympic Games context.
- Study populations involving Olympic athletes or stakeholders (eg, spectators, volunteers, and officials).
- Focus on health risks (eg, illness and injury) or safety risks (eg, terrorism).
- All publication types with accessible full text.
- Papers written in English.

Exclusion criteria

- Studies not related to the Olympic Games.
- Case reports or studies on individual athletes without group-level analysis.
- Studies unrelated to illness, injury, and terrorism, or imaging detection studies.
- Publications with unavailable full text.
- Paper not written in English.

### Data Extraction

General information was extracted (ie, year of publication and type of study). Data related to the characteristics of the Summer Paralympic Games, the Winter Paralympic Games (ie, date, location, type of study group, illnessnesses, injury, or terrorism studied, etc), was extracted, along with the key findings of each study, including the occurrence of illnesses, injuries, terrorist attacks, and so forth, as well as preventive measures (Multimedia Appendix 1).

### Classification of Illnesses

The classification of illnesses in this study will be the IOC consensus statement: methods for recording and reporting of epidemiological data on injury and illness in Sport 2020 (including STROBE [STrengthening the Reporting of OBservational studies in Epidemiology] Extension for Sport Injury and Illness Surveillance) [88] were combined with the *International Classification of Diseases* [89], which was processed and fused to form an innovative classification of illnesses applicable to this review and eventually divided into 18 organ or system classifications (Multimedia Appendix 1).

## Results

### General Characteristics of Included Studies

**Figure 1.**
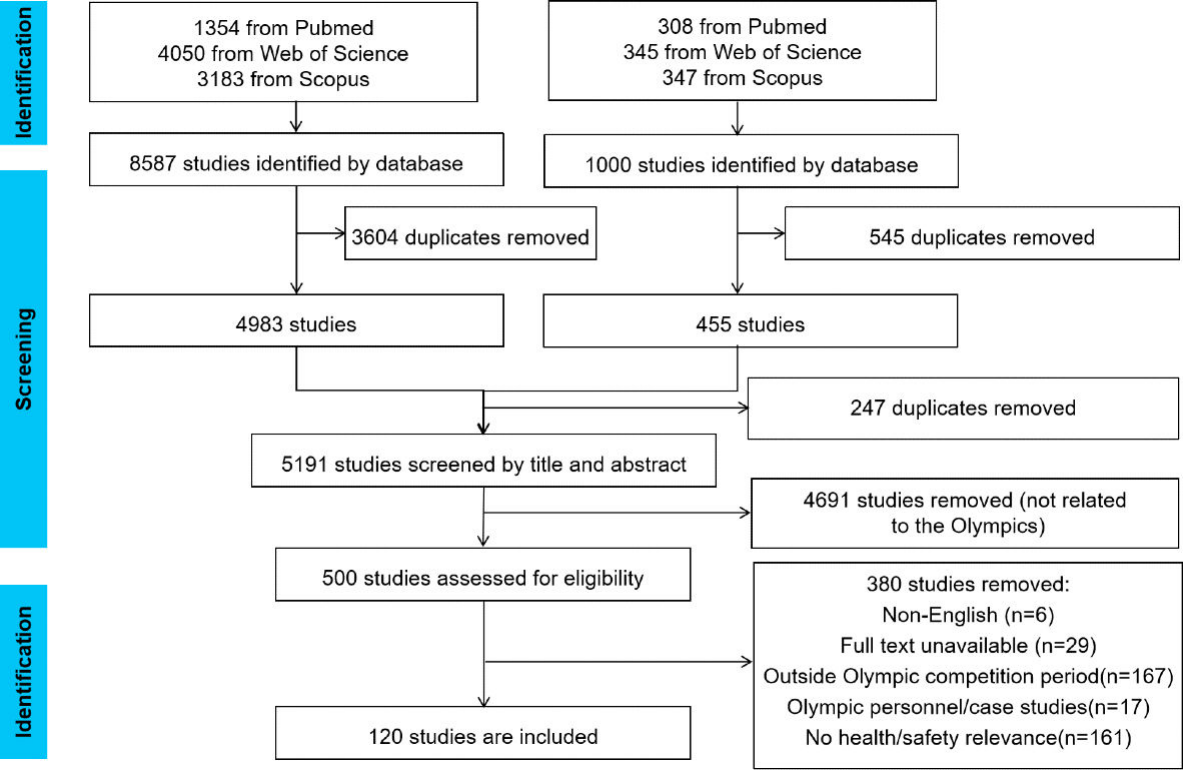
Screening process for studies eligible for inclusion. The first search yielded 8587 studies, and the supplementary search yielded 1000 studies.

We searched the database, and the first search included 8587 studies, which were supplemented for the Olympic infectious illness studies, and 1000 were searched again, and 120 studies were contained after removing duplicates and screening (Figure 1).

**Figure 2.**
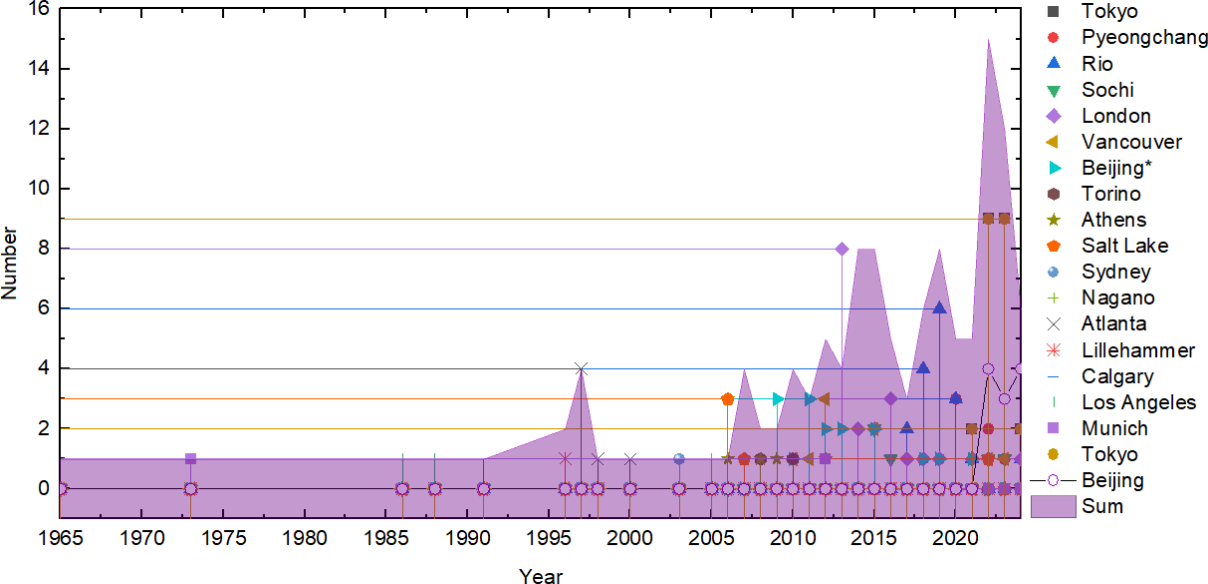
Trends in the number of studies per Olympics and the number of publications per year in the literature (shaded). Beijing refers to the 2022 Beijing Winter Olympics, and Beijing* refers to the 2008 Beijing Olympics.

Only 12 of the 120 studies on the Summer, Winter, and Paralympic Games were published before 2000, while 108 appeared from 2000 onward, reflecting an unprecedented surge in this field of research, particularly in recent years. Eighty-one of the 120 studies focused on illness, 81 on injury, and 2 on terrorism. Of the 120 studies, 18 analyzed events such as weight lifting and gymnastics; 9 analyzed bodily parts including shoulders, feet, and dentistry; and 26 analyzed particular illnesses or injuries such as COVID-19 disease, heat-related illnesses, and concussions, including 11 on COVID-19 disease. Out of all the studies conducted, 79 focused on the Summer Games, 48 on the Winter Games, and 50 on the Paralympic Games. Tokyo 2021, London 2012, and Rio 2016 were the top 3 most studied games (Figure 2). Across all illness studies, the most common organ or system illness for each study based on illness organ or system classification was respiratory system illness (n=35), the second most common organ or system illnesses for each study were gastrointestinal system illness (n=17) and skin system illness (n=8), respectively, and the third most common organ or system illnesses for each study were gastrointestinal system illness (n=10) and skin system illness (n=7). Additionally, the 120 publications included 3 reviews on injuries in Paralympic sports, 1 review on injuries in Winter Paralympic sports, 1 review on injuries in Winter Olympic Games, and 1 review on terrorist attacks.

### Health and Safety Risks and Influencing Factors

#### Risk and Influencing Factors of Illnesses

##### Respiratory System Illness

Respiratory illnesses have been the most common illnesses at all Olympic Games, and the IOC Medical and Scientific Committee has noted that acute respiratory illnesses are often the most common reason for athletes to seek medical assistance at major sporting events [90]. The number of respiratory infections during the 1996 Summer Olympics in Atlanta [91] and the overall number of respiratory illnesses at the 2008 Beijing Olympics [92] reached a peak of 922 and 3944 cases, respectively. The system most affected by illness during the London 2012 Paralympic Games was the respiratory system [15]. COVID-19 disease has also caused some spread in events such as the Olympics. For example, the Tokyo Olympic Committee recorded 430 cases of infections related to the Olympic Games [93]. The total number of positive cases monitored under closed-loop management during the Beijing Winter Olympics and Paralympics was 212 [94].

The primary determinants of respiratory illnesses are infection, allergies, environment, and various other related factors. Viruses and bacteria, such as measles, chicken pox, and influenza, primarily cause infections and widely affect the respiratory system. For example, respiratory illnesses were mostly caused by infections in the London 2012 Paralympics [95], Sochi 2014 Winter Olympics [96], Rio 2016 Olympic Games [97], and Pyongyang 2018 Winter Olympics [98]. The main risk factors for allergy and the environment are the mechanical and dehydrating pressures generated in the airways and the levels of air pollutants, irritants, and allergens inhaled by athletes during hyperventilated exercise conditions [99]. Symptoms of pollen allergy, such as worsening rhinoconjunctivitis and asthma, can be destructive to athletes expecting their best performance [100]. Prolonged exposure to mixed exhaust aerosols during exercise may result in reduced lung function and may promote vascular dysfunction [101]. During the Winter Olympics, physical activity in cold and dry air conditions causes inflammation and damage to the airways, possibly leading to a higher incidence of hyperresponsiveness to airways and asthma [102]. The long period of preparation and hard work during the 2008 Beijing Olympic and Paralympic Games led to the prevalence of other factors, such as fatigue, in staff respiratory illnesses [92].

##### Gastrointestinal System Illness

Illnesses of the gastrointestinal system are widely affecting athletes and those associated with the Olympic Games. Some studies have found that competitions, such as running, are the most demanding type of exercise for the gastrointestinal tract. More than 80% of athletes encounter gastrointestinal issues while exercising [103]. There were 120 cases of illnesses in the gastrointestinal system during the 2012 Summer Olympics in London, which accounted for 16% of all illnesses [104]. There were 131 cases of gastrointestinal system illnesses at the Rio 2016 Olympics [7]. At the 2018 PyeongChang Winter Olympics, 168 staff members were diagnosed with norovirus at the team’s accommodation near PyeongChang, and 4 athletes were also detected with the virus [105].

The primary determinants of gastrointestinal system disorders encompass infection, food, environment, and other related factors. In terms of infection, about 90% of viral gastroenteritis outbreaks are caused by norovirus [106]. The majority of virus transmission occurrences happen through person-to-person contact, contact with infected surfaces or environments, ingestion of food, or ingestion of water [107]. Allergies, environmental pollutants, and vigorous physical activity also contribute to the development of gastrointestinal illnesses [95,108]. Between 30% and 50% of people who play power endurance sports may have 1 or more problems with their stomachs, such as diarrhea, nausea, vomiting, and heartburn [108]. It has also been shown that variables such as gastrointestinal symptoms at rest, age, gender, diet, and years of training are associated with gastrointestinal discomfort during exercise [109].

##### Skin System Illness

Olympic athletes are susceptible to traumatic, environmental, and infectious skin disorders [110]. At the 1996 Atlanta Olympic Village polyclinic, 261 cases of dermatological conditions were documented, including rashes, infections, and other skin-related issues [111]. During the Tokyo 2020 Paralympic Games, there were 73 cases of skin illnesses, accounting for 26.1% (73/280) of all illnesses [112]. Skin and subcutaneous tissue illnesses occurred in 91 cases during the London 2012 Paralympic Games and are the second most common illnesses [113]. During the PyeongChang 2018 Paralympic Winter Games, skin system disorders such as limb or prosthetic interface lesions, residual limb or skin subcutaneous pain, rashes, skin breakdown, and abscesses were observed [114]. Skin system illnesses were the third most common system illness during the Beijing 2022 Paralympic and Paralympic Winter Games [5,14].

The main influences on skin system illnesses are infections, high-intensity physical activity, and specific competitions [115]. Skin system illnesses are particularly common among Paralympic athletes. Causes of skin infections in athletes with spinal cord injuries, amputations or limb deficiencies, and so forth, include loss of sensation and prolonged exposure to pressure in wheelchair-bound athletes, high forces at the residual limb-socket interface, hot or humid environments, sweating during exercise, and possible bacterial contamination in the exercise environment [95]. Skin and subcutaneous tissue are the most commonly affected systems in wheelchair basketball, weight lifting, and seated volleyball [113]. Certain sports are particularly prone to frictional stresses caused by repetitive movements, such as jogging, racquetball, baseball, golf, or any sport that entails frequent contact with equipment [115].

##### Heat-Related Illness

Heat-related illnesses affect a significant number of athletes and Olympic professionals at the Summer Olympic Games. A total of 213 heat-related illnesses were reported at the 1984 Los Angeles Olympics [116]; 372 people were treated for heat-related illnesses at the 1996 Atlanta Olympics, with symptoms including heat cramps, dehydration, heat syncope, and heatstroke [117]; at the 2008 Beijing Olympics, during the opening ceremony alone, there were up to 381 cases of heat-related illnesses [118]; and 225 cases of heatstroke occurred during the 2021 Tokyo Olympics, of which 100 were treated in athlete clinics and 125 in spectator clinics [119]. Therefore, it is evident that a rise in temperatures and other environmental factors during the Summer Olympic Games will result in heat-related illnesses among a significant number of people.

**Figure 3.**
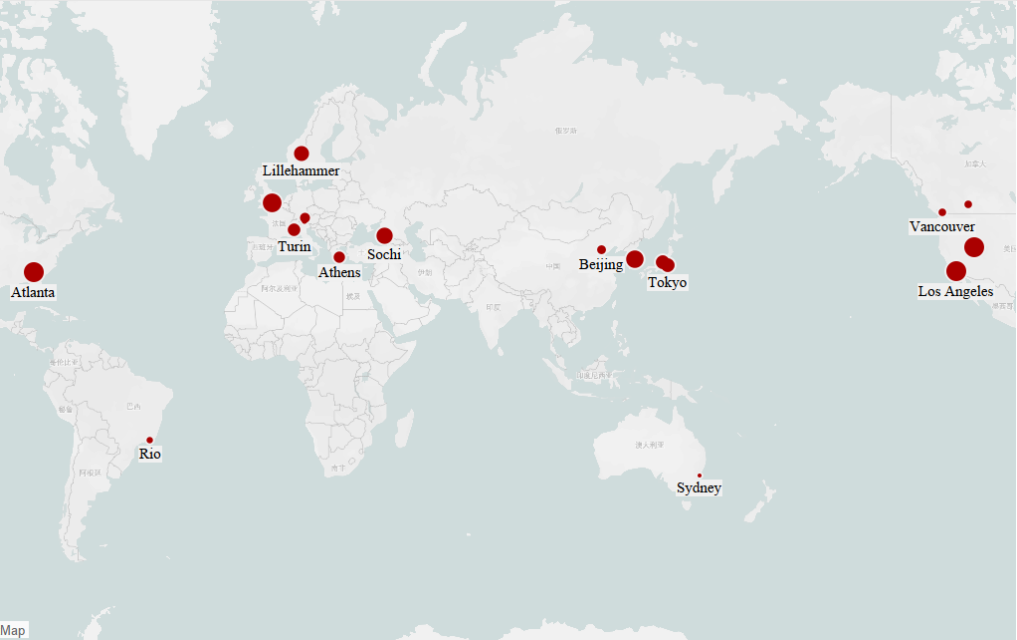
Geospatial location of the Olympic host cities included in the study.

Factors contributing to mass heat-related illnesses are the environment, the specific competition, and the climate of the host country, with climate being the dominant factor. Spectators in hot outdoor environments, among others, account for the majority of heat-related illness visits (939/1056, 88.9%), while members of the Olympic family are largely indoors in cooler temperatures, where heat-related illnesses are rare [91]. A study pointed out that marathon, race walking, cycling, and competitive swimming are the Olympic sports with the highest frequency of heatstroke illnesses [120]. The climate of Atlanta, which is expected to pose a significant risk for heat-related illnesses [120], is consistent with this study, showing that the climate of the host country of an event plays a determining role in the occurrence of heat-related illnesses. As an example, Tokyo experiences a tropical monsoon climate, Beijing experiences a temperate monsoon climate, and Los Angeles experiences a Mediterranean climate (Figure 3). The shared features of the Olympic Games held in these countries are high temperatures, which directly contribute to the prevalence of heat-related illnesses.

##### Other Organ or System Illness

**Figure 4.**
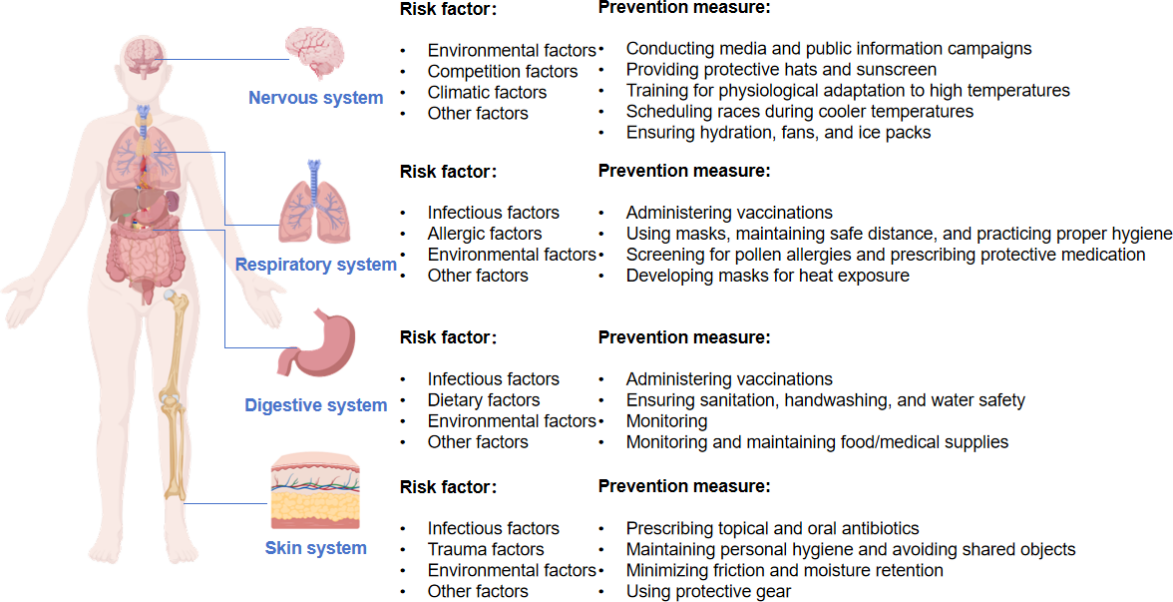
Factors affecting high-risk illness and preventive measures in the Olympic Games. Figure 4 is an original image created by the authors; however, the organ images were sourced from BioGDP [127].

Furthermore, there was a higher prevalence of neurological illnesses, genitourinary illnesses, and circulation illnesses. A total of 38 neurological illnesses were recorded at the 2016 Rio Olympics, representing 6% of all illnesses [7]. At the opening ceremony of the 2008 Beijing Olympics, a total of 25 instances of urinary tract illnesses were observed [118]. The 2008 Beijing Olympics and Paralympics reported a total of 49 instances of cardiac illness and 34 instances of vascular illnesses [121]. In addition to this, psychological issues such as competition stress and mental illness in the Olympic Games, especially the Paralympic Games, should also be given attention [111,122]. Special consideration should be given to illnesses associated with colds in susceptible populations participating in the Winter Olympic and Paralympic Games [123]. There have been numerous fatalities at the Olympic Games, with 1 athlete in each of the snowboarding events in 1964 and 2010 losing their life [124,125], a cameraman dying of cardiac arrest during the 1996 Olympic Games [91], a doctor from the Alpine Ski Team accidentally colliding with a tracked snowmobile and dying instantly in 2012 [123], and an athlete at the 2016 Summer Paralympic Games in Rio who experienced a head injury during the competition (Figure 4) [126].

### Risk and Influencing Factors of Injuries

Information on injuries related to the Summer and Winter Olympic Games is given in Table 1. Alpine skiing, Nordic skiing, and sledge hockey are the 3 key sports in the Winter Paralympics, and their associated sports injuries are more prominent [122]. Compared with recreational sports, the Olympics are faster, higher, and more competitive, with athletes having greater difficulty completing competitions and often neglecting minor injuries for the sake of winning honors, which can lead to more serious injuries [128,129]. The most common areas of sports injuries in the Summer Olympics are focused on the lower limbs and lower back, which may be related to the fact that the lower limbs and lower back need to bear the weight and impact of the human body in high-speed running, change of direction, and jumping, as well as high confrontation and high-load states, and are prone to injuries [130]. The most common types of injuries in Winter Olympic Games may be related to the movements executed in these sports, many of which involve high-speed twisting and shear forces [128]. For instance, ice hockey, a sport included in the Winter Olympics, is a sport marked by fast movements and intense body contact, resulting in contusions and sprains in vulnerable anatomical regions such as the hands [131]. Paralympic athletes experience a higher frequency of injuries to their upper extremities, namely, the shoulder, wrist, and elbow. This is because wheelchair athletes heavily depend on their shoulders for all their activities. Additionally, the nature of the injuries experienced by some of these athletes results in a smaller number of athletes participating in competitions with fully functioning lower extremities compared with their upper extremities [132]. The effect of competition difficulty, protective equipment, and assistive devices on injury has been noted in Winter Paralympic injury research [122]. Compared with summer sports, winter sports have a higher incidence of contusions, fractures, and concussions due to impact and speed [133]. Additionally, the Olympic Games have introduced new sports, posing challenges to precompetition and in-competition prevention and treatment efforts [129].

**Table 1.** Information on injuries related to the Summer and Winter Olympic Games.

Games	Highest risk competitions	Most common areas of injury	Most common types of injuries
Summer Olympics [130]	BMX, taekwondo, football, rugby, mountain biking, boxing, handball, hockey, weight lifting, etc	Knee, thigh, ankle, lumbar spine, calf, and foot	Joint sprains or ligament ruptures, muscle strains, contusions or hematomas, skin lacerations, and tendinopathies
Winter Olympics [131]	Freestyle skiing, snowboarding, alpine skiing, bobsledding, and ice hockey	Knees, chest/waist/back, and wrists/hands/fingers	Contusions, hematomas, and bruises, followed by strains (including muscle breaks, tears, or tendon ruptures) and sprains (including dislocations, subluxations, and ligament ruptures)

### Risk and Influencing Factors of Terrorist Attacks

The Olympic Games are highly susceptible to terrorist groups due to their prominent presence, the magnitude of the events, and the significant number of spectators. On September 5, 1972, a group of 11 Israeli athletes and coaches were abducted and subsequently murdered by members of the Black September organization at the Athletes’ Village [18], and during the 1996 Olympics in Atlanta, the 100-year anniversary of the Olympic Park bombing killed 2 people and sent 111 victims to city hospitals [17]. Besides terrorist acts that took place immediately during the Olympic Games, there have been other terrorist occurrences that are either directly or indirectly associated with the Olympic Games or other significant athletic events. Following the news of London’s successful attempt to host the 2012 Olympic Games on July 7, 2005, 4 terrorist assaults occurred in the city within 24 hours, resulting in the loss of 52 lives. On the eve of the 2010 World Cup final, an al-Qaeda spokesman declared on a North African jihadist website that the tournament would be targeted by suicide bombers using “undetectable explosives” [18]. In a study that noted at least 74 terrorist attacks against stadiums in the last 50 years, there have been 4 or more attacks in each subsequent year since 2011, with a peak of 11 in 2015 [134]. In recent years, although there have been no terrorist attacks at Olympic Games events, the Games may face other emergencies, such as demonstrations, bombings, and so forth, including bioterrorist attacks.

### Illness, Injury, and Terrorism Risk Surveillance System

Injury and illness surveillance at major sporting events prior to the 1990s relied primarily on traditional paper records and manual counting [135]. Actually, this appears to be the situation with the monitoring of the Olympic Games. With the rise of the internet and information technology, injury surveillance systems have gone through the following 3 phases.

The stage of information technology attempts was roughly from the 1980s to the early 2000s. With the rise of computer and internet technology, the Olympic Games began to experiment with the application of information technology to injury and illness surveillance, recording athletes’ health status through a preliminary surveillance network system or an electronic medical record system, and using databases for data storage and management. For example, the active illness surveillance system during the 1984 Summer Olympics in Los Angeles included a network of 46 hospitals, 90 doctors’ offices, and so forth [136]. Two complementary public health surveillance systems, the Georgia Department of Human Resources Sentinel Hospital System and the Atlanta Commission for the Olympic Games Health Information System, were established for the 1996 Summer Olympic Games in Atlanta [117]. Early surveillance methods still do not have convenient and efficient features. They are achieved through the employment of workers and fixed weekly calls with the match venue to achieve the purpose of surveillance, such as 4 levels of detection of injury classification scale, in order for the researchers of the charts to be retrospectively reviewed in order to understand the patient’s body-related situation [136,137] .

The networking phase was roughly from the beginning of the 21st century to around 2010. With the spread and development of internet technology, the Olympics began to establish a web-based injury and illness surveillance system. These technologies provide instantaneous transmission and exchange of data to enhance the effectiveness and precision of surveillance mechanisms. For the Sydney 2000 Olympic Games, public health planning covering disaster response planning, environmental hazard monitoring, and food safety strategies was developed, as well as surveillance systems covering both domestic and international parts of the country, including enhanced infectious illness surveillance, bioterrorism monitoring, global outbreak intelligence, and so forth [138,139]. The 2002 Salt Lake Winter Olympics developed a new public health surveillance system based on electronic medical records, with the goal of identifying unusual patterns of symptoms that could represent clusters of illness, with the hope of identifying these events of significance in a timely manner so as to allow an adequate public health response and management [140]. The International Paralympic Committee implemented the injury surveillance system and continued to do so at all subsequent Winter Paralympic Games [141]. The Athens 2004 Olympic Games used a team sports injury-reporting system, which reported information such as the injured player’s jersey number, time of game, site of injury, and type of injury [142]. The 2008 Beijing Olympics used a medical intake system to record unusual patterns of illness or injury that required further investigation [92].

**Figure 5.**
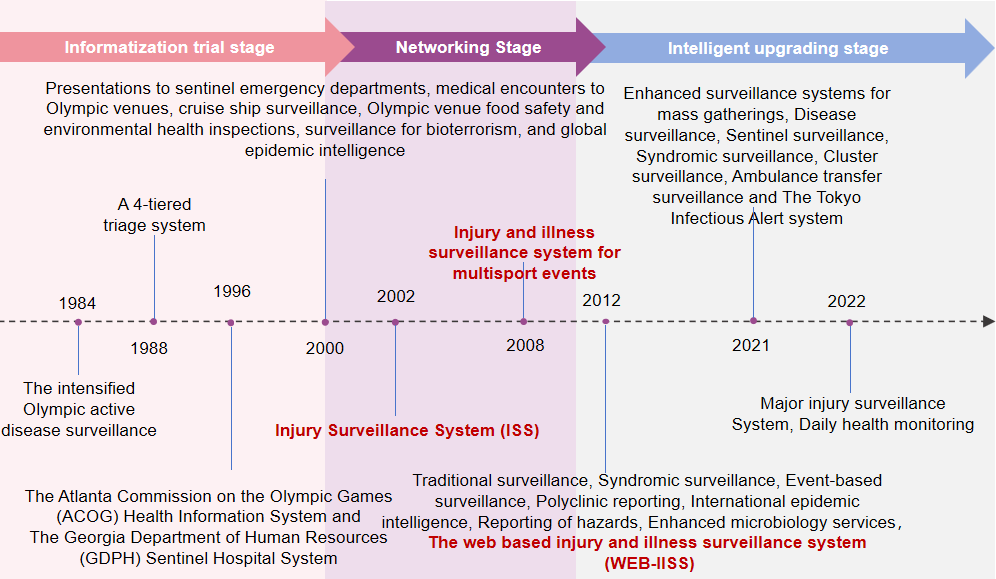
Main surveillance systems for injury and illness at previous Olympic Games.

The intelligent enhancement stage is mainly from 2010 to the present. With the rapid development of big data, artificial intelligence, and other technologies, the Olympic Games’ web-based injury and illness surveillance system has been continuously upgraded and improved, and the system is able to use data analysis technology to dig out the rules and trends behind the data, providing a scientific basis for the formulation of prevention and control measures (Figure 5). London 2012 Olympic and Paralympic Games used traditional monitoring, integrated monitoring, event-based surveillance, integrated clinic reporting, and so forth [16], with escalating scientific and technological methods. The IOC’s injury and illness surveillance system for multisport events was used in the Olympic Games in Beijing 2008, London 2012, Sochi 2014, Rio 2016, and Pyeongchang 2018 [96-98,143]. The intelligence drive led to the first adoption of the Web-based Injury and Illness Surveillance System at the London 2012 Paralympic Games, and since then, the WEB-IISS has been adopted and continually improved and enhanced at the Sochi, Rio, PyeongChang, Tokyo, and other Paralympic Games [112,114,132,144,145]. Surveillance for the Tokyo 2021 Olympic Games also involves multiagency and multifaceted aspects, including Japan’s Extreme Emergency and Post-Disaster Surveillance, Open-Source Epidemic Intelligence, and Multi-Surveillance System [119,146,147]. Injury surveillance for the Beijing 2022 Winter Olympic Games remains an integral part of the IOC’s initiative to protect the health of athletes and record injuries and illnesses in a web-based tabular format, as well as data from the local organizing committee’s medical (polyclinic) facilities and on-site medical support [14,119].

## Discussion

### General Characteristics

There were only 12 studies on the Summer Olympics, Winter Olympics, and Paralympics before 2000, and a total of 108 studies published in 2000 and beyond, particularly in recent years when the amount of literature in this area of research reached an unprecedented level, reflecting the fact that in recent years the importance attached to health and safety issues at the Olympic Games has reached an unprecedented level. Furthermore, the 3 most extensively studied games also demonstrate the growing emphasis on health and safety in the games in recent years. The 120 studies involving comprehensive papers on injury and disease, single competitions, body parts, and single injuries or illnesses show that in addition to holistic studies of illness and injury, scholars are also focusing on high-risk competitions and major injury areas. It is noteworthy that only 2 studies were conducted on terrorism, reflecting, on the one hand, the theme of peace and development and the near absence of terrorism in recent years and, on the other hand, the lack of ability to think in peace and insufficient research on new types of bioterrorist attacks.

### Related Health Risks

Illness is the most common risk factor in Olympic events. Illnesses of the respiratory system, the gastrointestinal system, and the skin system should pay particular attention to the infectious factors, which can be prevented and controlled in terms of the sources of infection, the route of transmission, and the susceptible population. In controlling the source of infection, attention is paid to screening for incoming illnesses, early diagnosis and isolation, and tracking, surveillance, and, if necessary, isolation and observation of close contacts of patients [16,148]. Emphasis should also be placed on dynamic surveillance and early detection of global illnesses, such as open-source epidemiological intelligence and multisource surveillance systems for infectious diseases [146,147]. In terms of cutting off the routes of transmission, it is advocated to wash hands frequently, wear masks, and avoid touching the mouth, nose, and eyes with hands, and so forth, so as to reduce the chances of pathogens spreading through hand-to-hand contact [6]. Maintaining cleanliness and hygiene in living and competition environments, with regular disinfection and ventilation, thereby reduces the survival time of pathogens in the environment and decreases pathogen concentrations in enclosed spaces [14]. Pay special attention to food and drinking water hygiene, avoiding food sold by street vendors, following water safety recommendations, and so forth [149]. With regard to the protection of susceptible groups, timely inoculation of appropriate vaccines and popularization of knowledge on the prevention and treatment of infectious illnesses, special attention is paid to the older adults, children, pregnant women, and other vulnerable and highly sensitive groups, and the necessary protective measures are taken, such as reducing the number of outings and avoiding crowded places [150].

Furthermore, illnesses affecting the respiratory and gastrointestinal systems, among others, can be attributed to a range of potential influencing factors such as environmental conditions (temperature, humidity, altitude, pollution, and pollen), diet, and exposure to diverse cultures, populations, and pathogens [151]. Particular attention should therefore be paid to environmental challenges related to heat, air quality, and water quality [152]. The management of respiratory system illnesses resulting from high altitude or cold conditions, air pollution, and pollen allergies includes the use of heat and moisture exchange respiratory device masks, allergy screening, and the administration of preventive drugs for illness prevention and control [100,102]. Dietary and nutraceutical approaches to regulate the microbial, together with the avoidance of nonsteroidal anti-inflammatory medicines and high fructose meals, can effectively treat gastrointestinal system issues induced by intense aerobic activity [103,153]. For illnesses of the skin system, especially moisture, sweat, friction [110], UV exposure [154] of body parts of specific competition events, and (people with disabilities) special populations should be considered. As heat-related illnesses can cause a medical surge, athletes and spectators (especially children, older adults, and vulnerable groups with underlying medical conditions) should be cooled, protected from the sun, hydrated, and avoided from competing in the heat [155]. Attention is also drawn to the recommendations of the World Economic Forum’s Global Commission on Biodiversity Cities on urban greening and the installation of white reflective roofs and continuously shaded pavements [156]. Furthermore, apart from preventing the aforementioned illnesses, our upcoming studies should also prioritize the mental health of professional athletes [122,128,157]. The issue of cardiac arrest should be taken seriously as deaths have occurred in previous competitions and reference can be made to the Resuscitation Council of Great Britain’s best practice guidelines on cardiac arrest [158]. Vigilance against traditional high-risk [159] and emerging unpredictable infectious illnesses should also be an important measure in illness prevention.

The IOC strongly encourages National Olympic Committees and International Federations to protect the health of athletes and actively focus on injury prevention [160]. It has been suggested that changes in the incidence of injuries may be the result of changes in the composition of the Olympic program, changes in environmental factors (eg, venue or track design, rules of the game, or other factors), changes in the awareness of athletes and their medical staff of the importance of recognizing and reporting minor incidents, and the availability of training in the recording of injuries and illnesses [96,98]. For example, encouraging taekwondo athletes to use more head kicks and removing helmet protection for boxers have led to a high incidence of injuries in combat sports, with the removal of protective helmet protection tripling the overall risk of injuries [143]. Injuries are common in the Olympic Games, and risk factors for injury mechanisms in high-risk events, video analysis, and precise biomechanics factors for movement techniques are essential for better direct injury prevention strategies [128,160]. Difficult and high-speed races are highly prone to injuries, and among the measures to prevent injuries are changing the rules of the competition, strengthening protective measures, and optimizing the track. For example, at the Sochi and PyeongChang Winter Olympics, modifiable injury risk factors were identified: redesigning the track, increasing the number of training sessions, changing the start time of the competition, and so forth [161]. Meanwhile, in the training of medical personnel, emphasis should be placed on clarifying common types of injuries and emergency procedures, with targeted training on contusions, muscle breaks, and sprains, including those to the knee, thoracic spine, lumbar spine, back, and other parts of the body [131]. Some studies, however, have also found an inverse relationship between the size of the National Olympic Committee (NOC) and the risk of health problems, noting that athletes from the smallest NOCs have significantly higher prevalence rates than athletes from the largest NOCs [98]. This is because larger delegations, often from countries with well-developed exercise physiology and sports medicine communities, can provide more comprehensive medical care and closer medical follow-up during the Olympic flag period, and therefore the IOC and host countries should consider the allocation of medical resources as a whole, with the inclusion of multilingual triage nurses and doctors as a noteworthy area of concern in the allocation of medical resources [162].

The problem of terrorist attacks at the Olympic Games has a long history, but in recent years, strong initiatives such as huge investments, high-tech equipment for monitoring, and armed military forces for security have hardly been associated with rumors of terrorist attacks at the Olympic Games [98]. Medical staff instructed on bomb blast injuries, pharmacies stocked with therapeutic drugs, and prepared to hose down contaminated facilities prior to the Atlanta Olympics Games [111]. The Sydney Olympics also established a bioterrorism-monitoring system [139]. The Tokyo Olympics also organized a number of training events to prepare for possible mass casualties, such as terrorist attacks [163]. It appears that plans have been formed for acts of terrorism. The world’s unprecedented changes are accelerating, international political disputes and military conflicts are erupting at various points, religious and ethnic conflicts still exist, and the global development and security situation is intricate and complex, which also poses certain risks to the security of the Olympic Games. Emerging biotechnology has also increased the cost, difficulty, and impact of bioterrorism attacks, with a particular focus on physical and mental health. Because of the Olympic Games’ widespread global influence, bioterrorism is a prime target, so it is critical to increase surveillance and take preventative measures beforehand.

### Illness and Injury Surveillance System

Surveillance systems in the Olympic Games have progressed from traditional paper records and manual statistics to information technology, networking, and intelligence, which can better facilitate the IOC’s discovery of injury and illness prevalence, but there are still shortcomings such as lagging surveillance technology, limitations in the scope of surveillance (eg, lack of epidemiological data on athletes’ mental health [164] and little attention to female athletes in the epidemiological data on injuries and illnesses [165]), data standards lack of uniformity, unequal distribution of monitoring resources in delegations, equipment safety, and radiation risks. In the future, technological innovation will integrate cutting-edge information technologies such as Internet of Things and blockchain to enhance monitoring intelligence and automation, enabling real-time surveillance and rapid response. Concurrently, data sharing and utilization efforts will strengthen cross-departmental and interinstitutional collaboration to dismantle information silos while promoting open data sharing and effective utilization. In privacy protection, reasonable data use will be advanced while ensuring data security through reinforced measures including encryption and access controls, guaranteeing safety throughout data transmission, storage, and usage. Finally, monitoring effectiveness will adopt an integrated ecosystem approach to assess population-level infectious disease risks, forging synergies among medical and laboratory systems, validated tools, and essential human and financial resources [166].

### Limitations of the Study

There are 3 key limitations to this study. First, most current illness classifications are by body system, which then makes it difficult to explore the causes and influences that trigger illness when analyzing the literature. Second, the study is also constrained in comparing different competitions as many papers have used different approaches to illness and injury data collection and research reporting. Third, this study does not include papers written in languages other than English, and information on Olympic-related illnesses, injuries, and terrorism may be published in the host country’s native language.

### Conclusions

From the perspective of mass-gathering medicine, this study investigates the occurrence of illnesses, injuries, and terrorism in the Olympic Games, analyzes the real reasons behind them, and then proposes targeted preventive measures. Through this study, we found that respiratory system illnesses, gastrointestinal system illnesses, skin system illnesses, and so on, are more common, and at the same time, heat-related illnesses also affect the Olympic personnel involved in a larger way; fighting, cycling, and ball and snow sports are prone to crowd casualties, and injuries are mainly in the knee, thigh, ankle, chest, waist, back, and so on, and the common types of injuries are contusions, strains, and so on; and the advancement of biotechnology and the volatile international situation have made bioterrorism attacks possible. In terms of health and safety risk prevention, the focus should be on vaccine-preventable illnesses, while health education should be strengthened, and so on; the rules of the competition should be revised, protective equipment should be upgraded, medical resources should be rationally allocated, and so on; and bioterrorist attacks should also be given high attention. In addition, injury and illness surveillance systems should pay attention to technological innovation, resource sharing, and information confidentiality.

## Supplementary material

10.2196/66829 Multimedia Appendix 1 Search strategy and detailed study characteristics.

10.2196/66829 Checklist 1 PRISMA-ScR checklist.

## Abbreviations

IOC: International Olympic Committee
NOC: National Olympic Committee
STROBE: Strengthening the Reporting of Observational Studies in Epidemiology
