# Supplementary material for: Preparing for Potential Health and Safety Risks at the Olympic Games: Scoping Review
Source: JMIR Public Health Surveill. 2025 Sep 3;11:e66829. doi: 10.2196/66829 (PMC12407570; doi:10.2196/66829)
Supplement: Multimedia Appendix 1 [file publichealth-v11-e66829-s001.docx]

**Search Strategy**

| **Search Strategy** | |
| --- | --- |
| Pubmed | ((Olympic[Title/Abstract]) OR (Paralympic[Title/Abstract]) OR (“Olympic Winter Game”[Title/Abstract]))AND ((health[Title/Abstract]) OR (diseases[Title/Abstract]) OR (illness[Title/Abstract]) OR (infectious diseases[Title/Abstract]) OR (Communicable Disease[Title/Abstract]) OR (injuries[Title/Abstract]) OR (trauma[Title/Abstract]) OR (heat-related illness[Title/Abstract]) OR (cardiac arrest[Title/Abstract]) OR (terrorism[Title/Abstract])) |
| WOS | (TS=(Olympic) OR TS=(Paralympic) OR TS=(“Olympic Winter Games”)) AND (TS=(health) OR TS=(diseases) OR TS=(illness) OR TS=(infectious diseases) OR TS=(Communicable Disease) OR TS=(injuries) OR TS=(trauma) OR TS=(heat-related illness) OR TS=(cardiac arrest) OR TS=(terrorism)) |
| Scopus | (TITLE-ABS-KEY(Olympic) OR TITLE-ABS-KEY(Paralympic) OR TITLE-ABS-KEY(“Olympic Winter Games”) AND (TITLE-ABS-KEY(health) OR TITLE-ABS-KEY(diseases) OR TITLE-ABS-KEY(illness) OR TITLE-ABS-KEY(infectious diseases) OR TITLE-ABS-KEY(Communicable Disease) OR TITLE-ABS-KEY(injuries) OR TITLE-ABS-KEY(trauma) OR TITLE-ABS-KEY(heat-related illness) OR TITLE-ABS-KEY(cardiac arrest) OR TITLE-ABS-KEY(terrorism)) |

| **Search Strategy(supplementary)** | |
| --- | --- |
| Pubmed | ((Olympic[Title/Abstract]) OR (Paralympic[Title/Abstract]) OR (Olympic Winter Game[Title/Abstract])) AND ((“Pathogen Transmission”[Title/Abstract]) OR (“Transmission, Pathogen”[Title/Abstract]) OR (“Transmission, Infectious Disease”[Title/Abstract]) OR (“Infectious Disease Transmission”[Title/Abstract]) OR (“Communicable Disease Transmission”[Title/Abstract]) OR (“Infection Transmission”[Title/Abstract]) OR (“Transmission, Infection”[Title/Abstract]) OR (“Transmission of Infectious Disease”[Title/Abstract]) OR (“Horizontal Transmission of Infection”[Title/Abstract]) OR (“Infection Horizontal Transmission”[Title/Abstract]) OR (“Community Transmission”[Title/Abstract]) OR (“Community Transmissions”[Title/Abstract]) OR (“Community Spread”[Title/Abstract]) OR (“Person-to-Person Transmission”[Title/Abstract]) OR (“Person to Person Transmission”[Title/Abstract]) OR (“Transmission, Person-to-Person”[Title/Abstract]) OR (“Autochthonous Transmission”[Title/Abstract]) OR (“Transmission, Autochthonous”[Title/Abstract]) OR (“Close-Contact Transmission”[Title/Abstract]) OR (“Close Contact Transmission”[Title/Abstract]) OR (“Transmission, Close-Contact”[Title/Abstract]) OR (Infections[Mesh]) OR (Infection[Title/Abstract]) OR (Infestation[Title/Abstract]) OR (Infectious[Title/Abstract])) |
| WOS | ((TS=(Olympic) OR TS=(Paralympic) OR TS=(“Olympic Winter Games”)) AND (TI=(Infections OR Infection OR Infestation OR Infectious) OR AB=(Infections OR Infection OR Infestation OR Infectious) OR TI=(“Pathogen Transmission” OR “Transmission, Pathogen” OR “Transmission, Infectious Disease” OR “Infectious Disease Transmission” OR “Communicable Disease Transmission” OR “Infection Transmission” OR “Transmission, Infection” OR “Transmission of Infectious Disease” OR “Horizontal Transmission of Infection” OR “Infection Horizontal Transmission” OR “Community Transmission” OR “Community Transmissions” OR “Community Spread” OR “Person-to-Person Transmission” OR “Person to Person Transmission” OR “Transmission, Person-to-Person” OR “Autochthonous Transmission” OR “Transmission, Autochthonous” OR “Close-Contact Transmission” OR “Close Contact Transmission”OR “Transmission, Close-Contact”) OR AB=(“Pathogen Transmission” OR “Transmission, Pathogen” OR “Transmission, Infectious Disease” OR “Infectious Disease Transmission” OR “Communicable Disease Transmission” OR “Infection Transmission” OR “Transmission, Infection” OR “Transmission of Infectious Disease” OR “Horizontal Transmission of Infection” OR “Infection Horizontal Transmission” OR “Community Transmission” OR “Community Transmissions” OR “Community Spread” OR “Person-to-Person Transmission” OR “Person to Person Transmission” OR “Transmission, Person-to-Person” OR “Autochthonous Transmission” OR “Transmission, Autochthonous” OR “Close-Contact Transmission” OR “Close Contact Transmission” OR “Transmission, Close-Contact”)) |
| Scopus | (TITLE-ABS-KEY(Olympic) OR TITLE-ABS-KEY(Paralympic) OR TITLE-ABS-KEY(“Olympic Winter Games”)AND(TITLE-ABS-KEY(infections OR infection OR infestation OR infectious) OR TITLE-ABS-KEY(“Pathogen Transmission” OR “Transmission, Pathogen” OR “Transmission, Infectious Disease” OR “Infectious Disease Transmission” OR “Communicable Disease Transmission” OR “Infection Transmission” OR “Transmission, Infection” OR “Transmission of Infectious Disease” OR “Horizontal Transmission of Infection” OR “Infection Horizontal Transmission” OR “Community Transmission” OR “Community Transmissions” OR “Community Spread” OR “Person-to-Person Transmission” OR “Person to Person Transmission” OR “Transmission, Person-to-Person” OR “Autochthonous Transmission” OR “Transmission, Autochthonous” OR “Close-Contact Transmission” OR “Close Contact Transmission” OR “Transmission, Close-Contact”)) |

**Types of illnesses**

(1) Specific infectious or parasitic illnesses; (2) Circulatory system illnesses; (3) Dermatological system illnesses; (4) Endocrine system illnesses; (5) Genitourinary system illnesses; (6) gastrointestinal system illnesses; (7) Respiratory system illnesses; (8) Musculoskeletal system illnesses; (9) Haematology or haematopoietic organ illnesses; (10) Neurological system illnesses; (11) Heat-related illnesses: (ICD) injuries, poisonings, or other consequences resulting from external processes, (consensus) thermoregulatory systems; (12) disorders affecting mental, behavioral, or neurodevelopmental functions; (13) Otology; (14) Ophthalmology; (15) Dentistry; (16) Interconnected systems; (17) Unidentified or unspecified; (18) COVID-19.

| Risks, prevalence, preventive measures and surveillance modalities by study | | | | | | | | | | | | |
| --- | --- | --- | --- | --- | --- | --- | --- | --- | --- | --- | --- | --- |
| Title | Monitoring method | Illness occurrence ranking | | | COVID-19 | Disease prevention measures | Highest risk occurrence matches | Most common types of injuries | Most common areas of injury | Injury prevention measures | Terrorism and prevention | Fatalities or cardiac arrest |
|  |  | 1 | 2 | 3 |  |  |  |  |  |  |  |  |
| MEDICAL SERVICE DURING THE WINTER OLYMPIC GAMES.... .1964... INNSBRUCK, AUSTRIA |  | (7) Respiratory diseases: Most of the diseases are colds and flu. | (6) Digestive disorders: one suffered from stage III malaria due to a trip to the Congo five months earlier. |  |  |  | Bobsledding and men's downhill piste racing are the top two most dangerous winter sports. | Most of the injuries were also minor, such as bruises, small cracks and sprains. 31 patients were taken to the University Hospital, 22 of whom required surgery. |  |  |  | Death: One of the competitors in the bobsled race suffered multiple fractures of the facial bones, ribs, right upper and lower arm, ilium and base of the skull. He died the day after his injury from a ruptured aorta, which may have been secondary to the trauma. |
| Pattern of injuries and illnesses in the Malaysian Olympic team |  | (7) Respiratory diseases: 12 cases of upper respiratory tract infection pharyngitis. | (6) Diseases of the digestive system: 2 cases of gastroenteritis, 1 case of gastritis. | (3) Dermatologic system diseases: one abscess and two cases of dermatomycosis. Dermatomycosis is another condition for which athletes rarely seek medical attention unless the itching and scaling are severe and the damage is unsightly. |  |  | Swimming, track and field, field field hockey. | The most common injuries are contusions, tears and muscle strains. |  |  |  |  |
| Special event medical care: The 1984 Los Angeles Summer Olympics experience | Records of medical services. | (11) Heat-related illnesses: 213, the disease ranked first. | (6) Digestive disorders: 140 people with minor gastrointestinal disturbances, the second highest number of disorders. | (1) Certain infectious or parasitic diseases: minor infections 129. |  |  |  | Minor musculoskeletal and skin injuries (24.8%). These included sprains, strains, myalgias, contusions, tendonitis, blisters, burns, abrasions and other minor injuries. |  |  |  |  |
| Public health at the 1984 Summer Olympics: The Los Angeles County experience | 1. Los Angeles County has had an active disease surveillance system in place since 1981 that identifies disease outbreaks, describes disease patterns and characterizes unusual disease occurrences;  2. In order to facilitate timely reporting of disease, the Department of Health established a 24-hour, 7-day-a-week telephone "hotline" and published the telephone number in the Department of Health newsletter for use by the medical community six weeks prior to the start of the Olympic Games. | (11) Heat Related Illnesses: Five hundred and sixteen (516) cases of heat related illnesses were reported: 464 cases from Olympic reporting locations and 52 cases from other locations. The equestrian events received the most media attention for heat-related complaints, reporting that 46% (total visits = 117) of visits were due to heat. | (6) Digestive Diseases: During the 1984 Summer Olympics, 7 to 10 cases of acute gastroenteritis were reported at each site. |  |  | 1. Under this policy, unlicensed food operations can be closed immediately without the usual two-week wait for a court order; 2.Acute Infectious Disease Control Disease surveillance and control is the responsibility of the centrally located Acute Infectious Disease Control Unit, which consists of physicians, epidemiologists and public health nurses. Three main activities were selected to provide the most sensitive means of recognizing disease incidence during the Olympic Games: the development of disease surveillance systems (active and passive), the establishment of telephone "hotlines" and the formation of public health emergency response teams; 3. Recommendations to reduce the incidence of heat-related illnesses included frequent announcements through loudspeakers at the venues urging spectators to drink water and avoid sun exposure where possible. |  |  |  |  |  |  |
| Level of medical care required for mass gatherings: the XV Winter Olympic Games in Calgary, Canada | Shortly after the competition, the researchers conducted a retrospective review of the charts for patient identity, age, gender, site, final diagnosis, severity, and nature. | (7) Respiratory diseases: upper respiratory tract infections 678. | (7) Respiratory diseases: 89 for influenza. |  |  |  |  | 599 musculoskeletal trauma; 442 skin trauma. |  | Teams of physicians with expertise in ALS should be used in rural Alpine ski resorts, where the risk of high-energy trauma is greater and transport times are longer. In communities where modern ALS ambulance services already exist and transport times are shorter, fewer physicians and larger teams of paramedics can be used in urban rallies. |  |  |
| Prevention and management of heat-related illness among spectators and staff during the Olympic Games - Atlanta, July 6-23, 1996 | The Atlanta Commission on the Olympic Games (ACOG) Department of Health Care Services, the CDC, the Georgia Department of Human Resources (GDPH) Department of Public Health, and other local, state, and federal public health agencies designed and implemented two public health surveillance systems. | (11) Heat-related illnesses: ACOG Health Information System: Between July 6 and 23, 372 (12.8%) people were treated for heat-related illnesses, including heat cramps dehydration, heat syncope, and heat stroke; 10 people were hospitalized. However, during an evening event attended by approximately 135,000 people, 54 (50.5%) of the 107 medical events handled by physicians were heat-related. GDPH Sentinel Hospital System: Between July 7 and 23, a total of 156 people, or approximately 2% of the emergency room visits for selected surveillance diseases, visited GDPH Sentinel Hospitals for heat-related illnesses; 15 people required hospitalization. 15 persons required hospitalization. |  |  |  | 1. Measures to prevent heat-related illnesses include wearing loose-fitting, light-colored clothing; wearing protective hats; increasing the intake of non-alcoholic beverages; maximizing time spent in air-conditioned environments; and staying in the shade inside and outside the venue. Whether the event itself is indoors or outdoors, spectators, staff, and others should take these precautions when they anticipate spending time outdoors (e.g., on their way to or from the event). Employers and supervisors should consider these precautions when establishing work schedules and rest periods for paid staff and volunteers;  2. Revise environmental health regulations to require the provision of free drinking water at events with more than 50 people, and implement aggressive media and public information campaigns. In addition, local government agencies and volunteer organizations have partnered to establish facilities that provide water, protective hats and sunscreen;  3. free drinking water was redeployed to areas with significant incidence of heat-related illnesses. The Atlanta Olympic Committee also used the data to assess and plan medical services. |  |  |  |  |  |  |
| Medical and public health services at the 1996 Atlanta Olympic Games: An overview | 1. Surveillance systems were established to detect emerging infectious disease outbreaks and unusual patterns of illness and injury, and to measure health service utilization during the Olympic Games;  2. the pre-existing passive notification system for infectious and other major diseases was enhanced with (1) active monitoring of medical conditions at eight sentinel hospitals, with daily data transmitted electronically to the Department of Public Health. (2) Daily reports from the Georgia Public Health Laboratory and the state's busiest private laboratory. (3) Physicians and other health care providers are encouraged to report unusual medical conditions directly to the state Department of Public Health. | (11) Heat-related illnesses: During the Games, more than 30,000 people sought medical assistance at first aid stations and clinics, of which 10,723 were examined by doctors. 10% were diagnosed with heat-related illnesses. For example, the highest rates of heat-related illnesses were recorded at the Beach Volleyball Courts (24 cases, no participants) and the Equestrian Park (19.7 cases, no participants). | (16) Multiple systems: The busiest medical facility was the polyclinic in the Olympic Village, where 2,474 Olympic staff and athletes were examined, many of them from other countries for routine dental and eye examinations. | (6) Digestive disorders: On the first day of the Olympic Games, two unlicensed food vendors were found in the Olympic Village, and two residents claimed that they had been linked to the onset of diarrheal diseases. |  | 1. Spectators were encouraged to drink plenty of water, seek shade and recognize symptoms of heat-related illnesses. In addition to an extensive media campaign, brochures were distributed to ticket buyers informing them of preventative measures. The Georgia Department of Public Health, the Red Cross and the Salvation Army joined forces to provide shelter, water, wide-brimmed hats, fans, sunscreen and prevention information to pedestrians along the corridors of the Olympic venues. In addition, water misters on high-speed evaporative fans were installed at 25 of the most crowded locations to help cool spectators and pedestrians. State health officials distributed guidelines to EMS providers and hospitals on recognizing and managing heat stroke;  2. physicians and public health workers launched a safe sex campaign to limit the spread of sexually transmitted diseases. Posters, pamphlets, and badges in 17 languages were used to convey the "safe sex" message, and 50,000 condoms in Olympic colors were distributed at polyclinics;  3. The city of Atlanta and the state of Georgia are vulnerable to natural disasters such as hurricanes and tornadoes; Hurricane Bertha threatened the Georgia coast a week before the Olympics, and the State Disaster Medical Response Team was placed on alert. Local agencies and organizations in Atlanta undertook extensive disaster planning efforts. In addition to revising their disaster plans, many healthcare organizations developed educational programs for responding to mass casualty events. More than 1,700 emergency room staff and prehospital personnel were trained to handle patients exposed to chemical, biological, or nuclear agents, and disaster drills were conducted. |  | Of these, 10,723 were examined by a doctor. The most common symptom examined by a doctor was injury (34%). |  |  | The bombing in Centennial Olympic Park on 27 July resulted in 2 deaths and 111 victims were transported to city hospitals. Most injuries were relatively minor, and only 24 victims required hospitalization.  Prevention: State officials, realizing that they lacked the resources to respond to a major terrorist incident, especially one involving chemical, biological or nuclear weapons, formally requested assistance from federal agencies, including the National Disaster Medical System. Urban Search and Rescue teams from across the U.S. were dispatched to Atlanta to assist in the rescue and treatment of victims of incidents such as building collapses. A five-member Disaster Medical Assistance Team was stationed at key locations around the city to provide rapid medical response. The Marine Corps deploys a 300-member, highly skilled chemical-biological incident response unit. Forensic and laboratory services were provided by units from the Federal Bureau of Investigation, the U.S. Army and U.S. Navy, the Environmental Protection Agency, and the Centers for Disease Control and Prevention. In addition, members of these units are stationed at CDC's specially convened Science and Technology Centers, which provide specialized public health and emergency medical, toxicological and scientific advice. | Fatalities: The July 27th Centennial Olympic Park bombing resulted in two fatalities. |
| Cardiovascular events in the 1996 Olympic Games |  | (7) Respiratory diseases: followed by acute upper respiratory tract infections (8.6%). | (11) Heat-related illnesses: heat cramps and dehydration (8%). | (2) Circulatory disorders: 136 (1.3%) had chest pain, 1.2% had hypertension, 0.6% had circulatory disorders, 0.1% had ischemic heart disease, and 0.01% had cerebrovascular events. Twenty-three Olympic-related patients were admitted to the three designated Olympic Family Hospitals with chest pain; eight were diagnosed with myocardial infarction and two subsequently underwent coronary angioplasty. Two others underwent coronary artery bypass surgery. |  |  |  | There were 10,723 medical consultations with doctors for Olympic-related patients. Of these, musculoskeletal sprains and strains accounted for 11.7% of incidents, contusions and abrasions (6.9%) and lacerations (4.4%). |  |  |  | Cardiac arrest: Two fatal cardiac arrests occurred. One occurred during the opening ceremony and involved the 54-year-old head of the Polish delegation. The medical team was attending to another patient nearby when they saw the man collapse. Cardiopulmonary resuscitation (CPR) was initiated within 15 seconds and defibrillation was completed within two minutes, but to no avail. Another cardiac arrest occurred in a 44-year-old Turkish photographer who collapsed in the excitement of the Centennial Park bombing and could not be resuscitated. |
| The Polyclinic at the 1996 Atlanta Olympic village |  | (3) Diseases of the skin system: 261 cases of skin diseases. | (7) Respiratory diseases: 200 cases of acute upper respiratory tract infections. | (11) Heat-related illnesses: Heat-related illnesses that usually occur during outdoor activities were uncommon (29 cases). |  |  |  | There were 396 sprains and strains and 156 contusions and abrasions. | Knee injuries were particularly common (about 70) and their incidence was comparable to the next three most common injuries (wrist, ankle and shoulder) . |  |  |  |
| Hospital use by Olympic athletes during the 1996 Atlanta Olympic Games |  |  |  |  |  |  |  | The most common was degenerative disc disease (4 cases), followed by patellar tendinitis and jaw fractures (2 cases each). |  |  | No athletes have attended since July 27, when an explosion at Centennial Olympic Park killed two people and injured 111 others. |  |
| Medical care delivery at the 1996 Olympic Games | 1. Health information systems are developed to monitor the health status of athletes, staff and spectators; to record service delivery; and to recognize unusual patterns of illness or injury that require further investigation;  2. For people with more serious problems treated by a physician, medical information is recorded on a standardized visit form;  3. every 2 hours, medical staff at each Olympic venue fax all record forms (from mobile rescue teams, sports medicine stations and spectator care stations) and all completed medical visit forms to a central location. The data from the log sheets and medical visit forms are entered into a computer, which analyzes the results and generates a daily report. | (7) Respiratory diseases: upper respiratory tract infections (n = 922, 9%) (e.g., 2 cases of malaria). | (11) Heat-Related Illnesses: Heat Cramps Dehydration (n = 801, 8%) Heat-related illnesses were the most common among spectators (n = 752, 21.6%). Spectators and volunteers accounted for the majority of heat-related illness visits (88.9%). Heat-related illnesses were rare among officials, members of the Olympic family, and the media. | (6) Digestive disorders: nausea and vomiting (n = 387, 4%). Among members of the Olympic family, upper respiratory tract infections were the most frequently listed reason for medical visits, and 2 chronic diseases, diabetes and hypertension, were among the 10 most common reasons for medical visits. |  |  |  | Sprains and strains were the most common reason for visits (n = 1450, 13%), contusions abrasions (n = 777, 7%), other injuries (n = 520, 5%), and lacerations (n = 487, 4%). |  |  |  | Cardiac arrest: 98 of the ill persons received emergency transportation for cardiac problems (i.e., chest pain, ischemic heart disease or high blood pressure). Three patients suffered cardiac arrest; of these, two were successfully resuscitated and transferred to hospital and one died. |
| Soft tissue injuries to USA Paralympians at the 1996 summer games | The USOC medical staff documented soft-tissue injuries that occurred during the 1996 Paralympic Games using the Sports Injury Reporting Form (SIRF) in the same grid as that used in the Olympic Health Insurance. |  |  |  |  |  |  |  | The most common injury sites for the specific four disability sports organizations were shoulder (26%), hip-thigh (14%), and ankle (12%) for the American Paralympic Association; hip-thigh (21%), cervico-thoracic region (19%), and shoulder (17%) for the American Association of Blind Athletes; lumbar (14%), toe (13%), and ankle (9%) for the United States Sports Association of Cerebral Palsy; and lumbar (14%), toe (13%), and ankle (9%) for the American Disabled Sports Association's shoulder (180o), arm-elbow (12%), forearm-wrist (12%), and low back (9%). | 1. Healthcare professionals assisting athletes with disabilities must be familiar with a wide range of pathologies and disability states;  2. the impact of a specific disability should be considered primarily in terms of how it affects movement and adaptations or assistive device stressors. Upper extremity joint protection should be a priority for athletes in the American Wheelchair Sports Association. Avoidance of overtraining and auditory-physical cueing responses should be a focus for athletes in the American Association of Blind Athletes, and lower extremity joint preservation, with an emphasis on conditioning the unimpaired lower extremity (lower extremity in athletes with unilateral pathology). |  |  |
| Watching the games: public health surveillance for the Sydney 2000 Olympic Games | Key components of the system include enhanced surveillance of infectious diseases; presentations to sentinel emergency rooms; medical contacts at Olympic venues; cruise ship monitoring; environmental and food safety inspections; bioterrorism surveillance; and global pandemic intelligence. Daily reports integrate data from all sources. | (7) Respiratory illnesses: On September 16, the day after the Opening Ceremony, the number of patients with upper and lower respiratory illnesses in Olympic medical facilities peaked at 90 per day, and then declined for the rest of the Olympic period. As of September 18, the number of influenza-like illnesses among residents of the Olympic Village had reached 28 cases. A review of these case records on September 19 showed that only three of these cases actually met the case definition of influenza-like illness. | (5) Diseases of the genitourinary system: 47 cases of sexually transmitted diseases and other genital diseases. |  |  | The incubation period of many infectious diseases means that cases of infection among Olympic visitors may not become apparent until after they have left. In retrospect, it would have been beneficial if emergency departments and other health-care facilities participating in the surveillance system had been equipped with rapid influenza diagnostic kits to improve the specificity of manifestations of "flu-like illness". |  | From the opening of the Olympic Village Polyclinic on September 3, 2000, to the close of operations on October 4, 2000, a total of 12,131 visits to medical facilities at the Olympic venues were recorded. One-third (36%) of the visits were for musculoskeletal problems, primarily for athletes, and 9.3% and 4.1% were for non-acute optometry and restorative dental procedures. |  |  | Prevention: This is important not only for containing the spread of influenza viruses, but also for detecting bioterrorism incidents, as the prodromal stages of many diseases caused by potential bioterrorism pathogens are difficult to distinguish from influenza. |  |
| Injuries among disabled athletes during the 2002 Winter Paralympic Games | 1 The International Paralympic Committee's Injury Surveillance System (ISS) was implemented during the 2002 Salt Lake City Winter Paralympic Games and has continued to be implemented at all subsequent Winter Paralympic Games;  2 Data for 36 injuries were recorded at the time of injury using the Injury Survey Instrument (ISI), while data for the other 3 injuries were obtained retrospectively using information recorded in the SLOC Medical Services Database. |  |  |  |  |  | Alpine skiers were the most frequently injured athletes with 24 injuries (62% of the total). Sled field hockey athletes ranked second in the total number of injuries, with 12 out of 39 injuries (31%), while Nordic skiers had the lowest number of injuries, with only 3 (8%) injuries. | The most common diagnoses were sprains (32%), fractures (21%), and strains and lacerations (14% each). |  |  |  |  |
| The 2004 olympic games: Physiotherapy services in the olympic village polyclinic | Documentation of injuries sustained by patients seen in the physical therapy department of a polyclinic. |  |  |  |  |  | The majority of injured athletes were from track and field (n = 169, 51.1%), followed by aquatic sports (n = 28, 8.4%) and weightlifting (n = 21, 6.3%). | Myofascial pain muscle spasms were the most common condition, followed by muscle strains, tendinopathies, and ligament sprains; in the nonathlete group, arthritis was the most frequent condition requiring physical therapy, followed by myofascial pain muscle spasms, root compression, and ligament sprains. | In the athlete group, the most common injury sites were the thigh, calf, knee and lumbar spine. In the non-athlete group, the lumbar spine, cervical spine and knees were the most common injury sites. |  |  |  |
| Hospital electronic medical record-based public health surveillance system deployed during the 2002 Winter Olympic Games | Hospital infection control professionals led a multidisciplinary team to develop a computerized rule-based system that relies on a patient's electronic medical record. | (7) Respiratory Diseases: Number of Influenza Cases in February 2002 (41 cases in total). |  |  |  |  |  |  |  |  |  |  |
| Torino 2006. XX Olympic and IX Paralympic Winter Games: the ENT experience |  | (7) Respiratory diseases: During the Olympic Games, 63% of tests were for acute upper respiratory tract inflammatory infections; during the Paralympic Winter Games, upper respiratory tract inflammatory infections accounted for 35% of diagnoses. | (13) Otology: During the Olympic Games, 23% were for acute otitis. During the Paralympic Winter Games, acute otitis accounted for 29%. |  |  |  |  |  |  |  |  |  |
| Sports injuries during the Summer Olympic Games 2008 | The attending physicians and or chief medical officers of the national teams were required to report all new injuries occurring during the Olympic Games on a standardized injury report form on a daily basis. In addition, physicians at the medical stations at the different Olympic venues and at the Olympic Village Polyclinic reported injuries on a daily basis. |  |  |  |  |  | Injury risk was highest in soccer, taekwondo, field hockey, handball, weightlifting, and boxing (all ≥ 15% of athletes), and lowest in sailing, canoeing kayaking, rowing, synchronized swimming, diving, fencing, and swimming. | The most common diagnoses are ankle sprains and thigh strains. |  |  |  |  |
| Analysis on the emergency medical services for the 2008 Beijing Olympic Games opening ceremony |  | (11) Heat-related diseases: The first-ranked diseases are heat-related diseases (381 persons, 66.49%). | (5) Diseases of the genitourinary system: diseases of the urinary system (25 persons, 4.36%). | (10) Diseases of the nervous system: followed by diseases of the nervous system (18 persons, 3.14%). |  |  |  | In second place were acute and chronic traumas (124, 21.64%). |  |  |  |  |
| Sports injuries and illnesses during the Winter Olympic Games 2010 | Require all National Olympic Committee (NOC) Chief Medical Officers to report new injuries and illnesses (or failures to occur) on a standardized reporting form on a daily basis. | (7) Respiratory diseases: a total of 113 cases (62.8%) of diseases affecting the respiratory system were found mainly in ice skating and Nordic skiing programs (Table 3). Therefore, the cause of the disease was most often categorized as infection (n=111, 63.8%), mainly affecting athletes in the aforementioned sports. | (7) Respiratory diseases: the most common diagnosis was upper respiratory tract infection (pharyngitis, sinusitis, tonsillitis) (n=61, 54.0%). |  |  |  | Snowmobiling, ice hockey, short track speed skating, alpine freestyle and snowboard cross-country have the highest risk of injury (15-35% of registered athletes affected in each sport). Nordic skiing (biathlon, cross-country skiing, ski jumping, Nordic biathlon), bobsledding, curling, speed skating, and freestyle snowmanship have the lowest risk of injury (less than 5% of registered athletes). | Contusions (31.6% females, 25.5% males), ligament sprains (19.8% females, 10.6% males), and muscle strains (8.1% females, 16.3% males) were the most common types of injuries. | The face, head and cervical spine (19.7% of females, 21.4% of males) and knees (16.1% of females, 10.7% of males) were the most commonly injured areas, followed by the wrists (8.0%) in females and the thighs (10.0%) in male athletes. |  |  | There has also been one catastrophic injury in bobsledding that resulted in a fatality. |
| Torino 2006 Winter Olympic Games: Highlight on health services organization | The organization consolidated the 18 medical centers operating in the Olympic venues, the three polyclinics in the Olympic Village, and the 13 existing Olympic hospitals. The design was a retrospective study that reviewed the medical records of the Olympic healthcare organizations. | (8) Diseases of the musculoskeletal system: The most common diagnoses belonged to the ICD9 subgroups "Injuries and poisoning" (although no cases of poisoning were recorded) and "Diseases of the musculoskeletal system and connective tissue", which together accounted for about 75% of the cases. | (6) Diseases of the digestive system: diseases of the digestive system (8.43%). | (7) Diseases of the respiratory system: diseases of the respiratory system (7.84%). |  |  | More than 25% of snowmobile (a variant of snowmobile and bobsled in which athletes must lie prone, facing downhill) athletes and nearly 20% of snowboarders require medical assistance, and similarly, snowmobile, alpine skiing, and freestyle skiing have higher rates of medical utilization than all other disciplines; for curling and short-track speed skating, athletes' rates of medical utilization are significantly lower than for all other disciplines; |  |  |  |  |  |
| Fit for the fight? Illnesses in the Norwegian team in the Vancouver Olympic Games | Disease prevalence data were obtained from the International Olympic Committee's daily injury and illness reports. | (7) Respiratory diseases: 4 out of 5 patients suffered from respiratory diseases. | (6) Digestive disorders: 1 person was diagnosed with mild enteritis. |  |  |  |  |  |  |  |  |  |
| Sonography in the 29th Olympic and Paralympic Games: A retrospective analysis | Ultrasound. | (17) Unknown or not specified: indications for ultrasonography included abdominal pain (315 cases, 41.50%). | (8) Diseases of the musculoskeletal system: Skeletal muscle diseases (228 cases, 30.04%). | (5) Diseases of the genitourinary system: gynecological diseases (104 cases, 13.70%). |  |  |  |  |  |  |  |  |
| Disease distribution and medical resources during the Beijing 2008 Olympic and Paralympic Games | A medical intake system was introduced to document unusual patterns of illness or injury that required further investigation. All patients were registered according to a preset standardized protocol in which personal data, initial diagnosis, type and time of accident, vital signs (heart rate, blood pressure, respiratory rate, and oxygenation), exercise, nationality, and function were recorded on a medical intake form. The name of the first responding physician is also recorded, as well as the type, date and time of medical treatment. | (7) Respiratory diseases: followed by respiratory diseases (Olympic Games, n=3944, 17.90%; Paralympic Games, n=1235, 15.35%); | (16) Multiple systems: ear, nose and throat diseases (Olympic Games, n=3507, 15.93%; Paralympic Games, n=1127, 14.01%). (11) Heat-related illnesses: Heat-related illnesses accounted for only a very small number of illnesses during the Olympic (n=594, 2.70%) and Paralympic (n=263, 3.27%) Games. |  |  |  |  | Surgical disorders were the most common reason for visits (Olympics, n=6211, 28.22%; Paralympics, n=3192, 39.67%), with fractures, ligament injuries, muscle injuries, maxillofacial trauma, friction burns, tendon strains, dislocations, traumatic injuries, arthritis, bursitis, tendonitis, foreign bodies, nerve entrapment, peroneal fascial compartment syndrome, and compound injuries. |  |  |  |  |
| Health protection of the Olympic athlete | 1 In Beijing and Vancouver, the chief medical officers of all National Olympic Committees participated in the Olympic Monitoring Study and reported new injuries and illnesses on a daily basis using a standardized reporting form (Beijing injuries only);  Injuries and illnesses of athletes seen at venue medical stations or central clinics were reported through the central clinic database. | (6) Digestive disorders: most (63%) affect the respiratory system. The most common diagnosis is upper respiratory tract infection (pharyngitis, sinusitis, tonsillitis; 54%). |  |  |  |  | Injury rates for different sports varied widely between Beijing and Vancouver. In Beijing, soccer, taekwondo, field hockey, handball, weightlifting and boxing had the highest injury risk (≥15% for all athletes). In Vancouver, bobsledding, ice hockey, short-track speed skating, alpine skiing and freestyle skiing and snowboarding cross-country had the highest risk of injury (15%-35% of registered athletes in each sport were affected). |  | In Beijing, the distribution of injuries was as follows: about half of the diagnoses (54%) involved the lower extremities, 20% the upper extremities, 13% the trunk, and 12% the head and neck. Thighs (13%) and knees (12%) were the most common sites of injury, followed by calf, ankle and head injuries (9%). In Vancouver, for both men and women, the face, head and cervical spine (20% of women and 21% of men) and knees (16% of women and 11% of men) were the most common injury sites. In alpine skiing, freestyle skiing and snowboarding, 22 of 102 injuries (22%) involved the head and cervical spine, and a quarter of injuries involved the knee (24%). Twenty concussions were reported, affecting 7% of registered athletes. |  |  | There was a fatality during the bobsled race. |
| Watched by the Games: Surveillance and security at the Olympics |  |  |  |  |  |  |  |  |  |  | 1. On 5 September 1972, 11 Israeli athletes and coaches were taken hostage in the Athletes' Village by members of the Black September organization, believed to be a militant faction of the Palestine Liberation Organization, the armed wing of Fatah, the political movement led by Yasser Arafat;  2. On Tuesday, September 11, 2001, Al Qaeda terrorists hijacked a number of airliners loaded with fuel, two of which intentionally crashed into the upper floors of the Twin Towers in New York City, causing the collapse of the towers and thousands of deaths and serious injuries, events that make the Black Sunday conspiracy seem less far-fetched. One could speculate that Al Qaeda chose the World Trade Center not only because it was packed with people, but also because it was widely recognized as an iconic symbol of America's global commercial domination. Similarly, major sporting events, such as the Olympic Games and the Soccer World Cup, not only gather large numbers of people, but are also highly symbolic of national prestige and power;  3. On July 7, 2005, it was announced that London had been successful in its bid to host the 2012 Olympic Games. Just 24 hours later, four terrorist attacks in London killed 52 people and changed the security landscape overnight;  4. In 2009, in Lahore, Pakistan, the Sri Lankan cricket team's motorcade bus was attacked by masked gunmen believed to be members of an extremist Islamist faction linked to Al-Qaida, who killed six policemen and the coach driver and injured several cricketers before being repelled by armed police. Less than a year later, in a similar incident during the African Cup of Nations, the Togolese national soccer team's bus was ambushed by guerrillas of the Front for the Liberation of the Enclave of Cabinda as it traveled through Angola's disputed province of Cabinda to the competition venue. Several team officials were killed and three players were seriously injured, casting a shadow not only over the Cup of Nations itself, but also over South Africa, which was to host the World Cup finals later that year;  5. on the eve of the 2010 World Cup finals, an al-Qaeda spokesman declared on a North African jihadist website that the tournament would be the target of suicide bombers using "undetectable explosives", which heightened the terrorist threat. the qualifier between England and the United States, played in Leustenburg on June 12, was specifically mentioned The USA vs England match [sic] was broadcast live in a stadium packed with spectators when explosions rumbled across the field, toppling the entire stadium and leaving the body count in the dozens to hundreds, Allah willing. (Edwards, 2010) The above statements made by the Al Qaeda organization (Al Qaeda) are low cost, but when compared to the history of indiscriminate and overt terrorist attacks carried out by the organization and its affiliates, including sports-related targets, they highlight the climate of fear that grips contemporary society. | Eleven Israeli athletes and coaches died. |
| Paralympic medical services for the 2010 paralympic winter games | All certified medical encounters at venues and polyclinics are entered by clinicians into the electronic medical record in the health system database. Each contact with a member of the medical service team is recorded as a contact. | (8) Diseases of the musculoskeletal system: the majority of medical errors during the Paralympic Games were musculoskeletal related (44.6%, n = 1156). |  |  |  |  |  |  |  |  |  |  |
| The injury experience at the 2010 winter paralympic games | Injury Questionnaire. |  |  |  |  |  | Sled field hockey had the highest incidence, with 40 injuries found among 118 athletes in these sports (IP = 33.9%). Alpine skiing had the second highest incidence, with 41 injuries found in 194 athletes (IP = 21.1%). |  |  |  |  |  |
| Illness and injury in athletes during the competition period at the London 2012 Paralympic Games: development and implementation of a web-based surveillance system (WEB-IISS) for team medical staff. development and implementation of a web-based surveillance system (WEB-IISS) for team medical staff | New Web-based Injury and Illness Surveillance System (WEB-IISS). | (7) Respiratory illnesses: A total of 365 athletes (10.2% (95% CI 9.2% to 11.2%) of the total number of athletes) reported 501 cases of illness during the competition. The system most affected by disease was the respiratory system (IP 27.5%; IR 3.6 (95% CI 3.0 to 4.2)). | (3) Dermatologic system diseases: followed by skin and subcutaneous tissue (IP 18.3%; IR 2.3 (95% CI 1.9 to 2.9)). | (6) Diseases of the digestive system: digestive system (IP 14.8%; IR 1.9 (95% CI 1.5 to 2.4)). |  |  |  |  | A total of 387 athletes reported 475 injuries. Shoulder injuries were the most common (83 injuries; 2.1/1000 athlete-days (95% CI 1.7 to 2.6)), followed by wrist and hand injuries (59 injuries; 1.5/1000 athlete-days (95% CI 1.1 to 1.9)), and elbow injuries (40 injuries; 1.0/1000 athletes (95% CI 0.7 to 1.4)). |  |  |  |
| Sports injuries and illnesses during the London Summer Olympic Games 2012 | Records were kept of (1) all National Olympic Committee (NOC) medical team reports and (2) daily occurrences (or lack thereof) of injuries and illnesses of London Organising Committee of the Olympic and Paralympic Games (LOCOG) medical staff at polyclinics and medical sites. | (7) Respiratory diseases: A total of 310 diseases (41%) affected the respiratory system, with the most common causative agent being infection (n=347, 46%). | (6) Digestive disorders: gastrointestinal (n=123, 16%). | (3) Diseases of the dermatologic system: skin diseases (n=83, 11%). |  |  | Athletes in taekwondo, soccer, scooter, handball, mountain biking, track and field, weightlifting, field hockey and badminton are at the highest risk of injury |  |  |  |  |  |
| Fit and healthy Paralympians - medical care guidelines for disabled athletes: a study of the injuries and illnesses incurred by the Polish Paralympic team in Beijing 2008 and London 2012 A study of the injuries and illnesses incurred by the Polish Paralympic team in Beijing 2008 and London 2012 | The Pre-Participation Examination (PPE) includes a general medical/orthopaedic examination, electrocardiogram, blood and urine tests. The mandatory Periodic Health Evaluation (PHE), introduced prior to the London Paralympic Games, included a general medical/orthopaedic/dental examination, anthropometric measurements, electrocardiogram, stress test, laryngeal and ophthalmologic consultations, and blood and urine tests. | (7) Respiratory diseases: Respiratory tract infections (RTIs) predominate in both PGs. |  |  |  |  |  |  |  |  |  |  |
| Factors associated with illness in athletes participating in the London 2012 Paralympic Games: a prospective cohort study involving 49,910 athlete- days | Daily disease data were obtained from (1) teams with their own medical support who completed daily disease logs on a novel web-based system (78 teams, 3329 athletes) and (2) teams without their own medical support via the local organizing committee database (82 teams, 236 athletes). | (7) Respiratory diseases: respiratory system (3.61). The highest prevalence of respiratory diseases was in cycling (road) (7.8), followed by table tennis (5.4), swimming (4.7) and athletics (4). | (3) Dermatologic system diseases: skin and subcutaneous tissue (2.40). Skin and subcutaneous disorders were most common in weightlifting (3.9), followed by wheelchair basketball (3.5) and table tennis (3.2). Skin and subcutaneous tissue were the most commonly affected systems in wheelchair basketball, weightlifting and seated volleyball. | (6) Digestive disorders: digestive system (1.90). Digestive disorders are most common in swimming (2.7), followed by athletics (2.7) and weightlifting (2.6). |  |  |  |  |  |  |  |  |
| The London 2012 summer Olympic games: An analysis of usage of the Olympic village 'Polyclinic' by competing athletes | Records of all types of visits were collected for analysis, and the figure combines medical consultations, radiology/pathology exams, and prescriptions. | (8) Diseases of the musculoskeletal system: Musculoskeletal division has the largest share (52%). | (15) Dental: Dentistry (30%). | (14) Ophthalmology: Ophthalmology (9%). |  |  |  |  |  |  |  |  |
| The epidemiology of injuries at the London 2012 Paralympic Games | 1. One database is derived from medical visit forms completed by medical providers during their assessment at one of the medical stations operated by the Organizing Committee. A second database was populated on a daily basis with information provided by team medical staff who completed a comprehensive web-based injury survey;  2. Of the 78 delegations that brought their own medical staff to the Games, 123 injuries were recorded in the LOCOG database and 458 injuries were recorded in the WEB-IISS. Of the 82 delegations that did not have access to their own medical support, 52 injuries were recorded in the EMDCS database and no injuries were recorded in WEB-IISS. |  |  |  |  |  | Futsal had the highest IR, followed by weightlifting, blind goalball, wheelchair fencing, wheelchair rugby, track and field, and judo. |  | The most common site of injury (as a percentage of all injuries) was the shoulder (17.7%), followed by the wrist/hand (11.4%), elbow (8.8%), and knee (7.9%). |  |  |  |
| The role of sports physiotherapy at the London 2012 Olympic Games | ATOS electronic medical record system for polyclinics. |  |  |  |  |  |  | Muscle (33.3%) and joint injuries (24.8%) were the most common diagnoses among athletes and non-athletes (24.4% and 30.1%). | The most commonly documented anatomical sites at the first visit for athletes were the knee (15.4%), lumbar spine/lower back (15.2%), and thighs (12.6%), and for nonathletes at the first visit, the most commonly documented anatomical sites were the lumbar spine/lower back (19.8%), knee (15.8%), and neck/cervical spine. |  |  |  |
| London 2012 olympic and paralympic games: public health surveillance and epidemiology | Traditional surveillance, integrated surveillance, event-based surveillance, integrated clinic reporting, international outbreak intelligence, etc. | (6) Diseases of the digestive system: mainly diseases related to gastroenteritis (possible food poisoning). |  |  |  |  |  |  |  |  |  |  |
| Descriptive epidemiology of paralympic sports injuries |  |  |  |  |  |  | The highest injury rates were in futsal, blind goalball, weightlifting, wheelchair fencing and wheelchair rugby |  |  |  |  |  |
| Injury risk in the olympic games: The injury and illness surveillance during the XXIX 2008 summer and the XXI 2010 winter olympic games | 1. Six months prior to the 2008 Beijing and 2010 Vancouver Olympic Games, the IOC notified the National Olympic Committees (NOCs) of the study. The medical representatives of all participating countries received a brochure detailing the study, including the injury and illness forms to be completed;  2. Injuries and illnesses of athletes attending medical stations at venues or central clinics were also reported through the central clinic database. In order to encourage compliance with reporting procedures during the Olympic Games, members of the study team maintained frequent personal contact with the National Olympic Committees of each country. |  |  |  |  |  | Beijing 2008 Olympic Games： soccer, taekwondo, field hockey, handball, weightlifting. | Beijing 2008 Olympic Games： Ankle sprain (7%), thigh strain (7%). | Beijing 2008 Olympic Games： Torso (13%), thighs (13%), head/neck (12%), knees (12%). |  |  |  |
|  |  | Vancouver 2010 Winter Olympic Games：(7) Respiratory diseases: The majority of diseases (n = 113, 63 %) affected the respiratory system, most commonly in the skating and Nordic skiing programs. Therefore, the cause of the diseases was most often categorized as infections (n = 111, 64 %), mainly affecting athletes in the above-mentioned sports. | Vancouver 2010 Winter Olympic Games：(7) Respiratory diseases: the most common diagnosis was upper respiratory tract infection (pharyngitis, sinusitis, tonsillitis) (n = 61, 54 %). |  |  |  | Vancouver 2010 Winter Olympic Games： snowboard cross-country, freestyle aerials and cross-country, bobsledding, ice hockey. | Vancouver 2010 Winter Olympic Games： Concussion (7%). | Vancouver 2010 Winter Olympic Games： Head/neck (16%), knees (14%), thighs (7%). |  |  | Vancouver 2010 Winter Olympic Games： A fatal injury occurred during a bobsled race. |
| Sports injury and illness epidemiology: Great Britain Olympic Team (TeamGB) surveillance during the Sochi 2014 Winter Olympic Games | The observational prospective cohort study followed the UK Injury/Illness Expression Project surveillance methodology and obtained information on injuries and illnesses in Team GB athletes (n=56) between January 30 and February 23, 2014. | (7) Respiratory diseases: Respiratory diseases are the most reported. |  |  |  |  | Freestyle skiing, snowmobiling and snowboarding have the highest risk of injury. | Sprains (joint/ligament injuries; 30%) and contusions (19%) were the most common types of injuries in the UK team. | The lower extremities are the most common site of injury. |  |  |  |
| Sports injuries and illnesses in the Sochi 2014 Olympic Winter Games | 1.Adoption of all National Olympic Committee (NOC) medical team reports; 2.Sochi 2014 medical staff reports at polyclinics and medical sites. | (7) Respiratory diseases: A total of 159 diseases (64%) affected the respiratory system. Infections were the most common cause of disease (n=145, 58%), mainly affecting athletes in the aforementioned sports. Of the 159 cases of respiratory diseases, 118 (74%) were caused by infections. | (6) Diseases of the digestive system: digestive system (n=28, 11%). | (10) Neurological disorders: neurological (n=13, 5%). |  |  | The highest injury rates were in aerial skiing (48.8 injuries per 100 athletes), snowboard slopestyle (37.0), snowboard cross-country (34.4), slopestyle skiing (30.8), U-course skiing (25.5), snow tricks skiing (24.6), alpine skiing (20.7), and snowboard halfpipe (18.2). | Twenty-five ligament sprains/ruptures, 14 fractures, six contusions, hematomas, or abrasions. | 23 cases affecting the knee. |  |  |  |
| Injury and illness surveillance among olympic athletes: Summary of the 2010 Winter, and the 2008 and 2012 summer olympic games | 1. The Chief Medical Officers of all NOCs participated in the Olympic Games Monitoring Study and reported daily on the occurrence (or lack thereof) of new injuries and illnesses (Beijing injuries only) using standardized reporting forms;  2. Injury and illness information for all Athletes treated by the Local Organizing Committee (LOC) medical services was obtained from the medical centers at the selected venues;  3. the Injury and Illness Report Form requires extensive documentation of the corresponding injuries and illnesses. A booklet was provided containing detailed instructions and examples of how to properly complete the form. Daily injury and illness information was also received from the Polyclinic in the Olympic Village. Injury and illness report forms were distributed to NOCs in all required languages. |  |  |  |  |  | Beijing 2008 Olympic Games： soccer, taekwondo, field hockey, handball, weightlifting. | Beijing 2008 Olympic Games： Ankle sprain (7%), thigh strain (7%). | Beijing 2008 Olympic Games： Torso (13%), thighs (13%), head/neck (12%), knees (12%). |  |  | Beijing 2008 Olympic Games： One person died in the bobsled race. |
|  |  | (7) Respiratory diseases: the most common diagnosis is upper respiratory tract infections (pharyngitis, sinusitis, tonsillitis). |  |  |  |  | Vancouver 2010 Winter Olympic Games, Highest risk of injury is in bobsledding, ice hockey, short track speed skating, alpine skiing, freestyle skiing and snowboarding cross-country. | Vancouver 2010 Winter Olympic Games： Concussion (7%). | Vancouver 2010 Winter Olympic Games： Head/neck (16%), knees (14%), thighs (7%). |  |  |  |
|  |  | (7) Respiratory diseases: the most common diagnosis is upper respiratory tract infections (pharyngitis, sinusitis, tonsillitis). |  |  |  |  | London 2012 Olympic Games: Highest risk: soccer, taekwondo, scooters, handball, mountain biking, track and field. | London 2012 Olympic Games: Shoulder, elbow and knee dislocations, muscle strains and ruptures, fractures/stress fractures, ligament sprains and ruptures (including ACL), tendon ruptures. |  |  |  |  |
| Risk of injuries in paralympic track and field differs by impairment and event discipline: a prospective cohort study at the London 2012 Paralympic Games | Shortly after the competition, the researchers conducted a retrospective review of the charts for patient identity, age, gender, site, final diagnosis, severity, and nature. | (17) Unknown or not specified: viral syndromes*, 808 cases. |  |  |  |  |  | Acute musculoskeletal trauma, skin trauma. |  |  |  | A deceased patient was an Alpine Ski Team physician who accidentally collided with a tracked snowmobile. The patient died at the scene, and the severity of his injuries prevented any intervention. |
| Paralympic sports medicine |  | (7) Respiratory Diseases: Respiratory System. 50% of respiratory diseases are thought to be caused by infections. Environmental conditions (including allergens and environmental pollution) account for 30% of respiratory diseases. | (3) Diseases of the Dermatologic System: Dermatologic system. 44% of skin and subcutaneous tissue diseases are thought to be caused by infections. Reasons for the higher incidence of skin infections include loss of sensation and prolonged exposure to stress in athletes who use wheelchairs. The residual limb-socket interface is also a high-risk area for amputee athletes because of exposure to high intensity, hot/humid environments, sweating during exercise, and possible bacterial contamination in the exercise environment. | (6) Digestive diseases: digestive system. Environmental conditions (including allergens and environmental pollution) account for 26% of digestive diseases. |  |  | The highest injury rates were in futsal, weightlifting, blind goalball, wheelchair fencing and wheelchair rugby. |  | The most affected anatomical regions in Paralympic sports (in descending order) include the shoulder, wrist and hand, elbow, knee, ankle and foot. |  |  |  |
| High incidence of injury at the Sochi 2014 Winter Paralympic Games: a prospective cohort study of 6564 athlete days | This study used the Web-based Injury and Illness Surveillance System (WEB-IISS), which was successfully implemented at the London 2012 Paralympic Games. Teams with their own medical support staff used WEB-IISS for data collection. For athletes without accompanying medical staff, injury data were obtained through the ATOS system provided by medical staff employed by the Sochi Organizing Committee for the Olympic and Paralympic Games (SOCOG). |  |  |  |  |  | Alpine skiing/snowboarding had the highest total number of injuries and reported IR (108 injuries in 81 athletes with an IR of 41.1. The second highest IR was in ice hockey, followed by wheelchair curling (and cross-country skiing/biathlon. |  | The most vulnerable part of the body is the upper limb, with 56 injuries occurring in 35 athletes. Forty-two of these injuries occurred in the shoulder. The incidence of injuries to the lower extremities was similar, with 55 injuries occurring in 45 athletes. Of these, the knee was the most vulnerable joint in the lower extremity.A total of 31 injuries occurred in the anatomical regions of the head, face, and neck in 26 athletes with an IR of 4.7.The CI for the IR of head, face, and neck injuries indicated that injuries to areas such as the head were higher than upper and lower extremity injuries were higher than all other anatomical regions. |  |  |  |
| Sports injury and illness incidence in the Rio de Janeiro 2016 Olympic Summer Games: a prospective study of 11274 athletes from 207 countries | The daily incidence of injuries and illnesses among athletes was documented (1) through reports from all National Olympic Committee (NOC) medical teams and (2) by Rio 2016 medical staff at polyclinics and medical sites. | (7) Diseases of the respiratory system: A total of 292 cases (47%) of diseases affected the respiratory system. Of the 292 respiratory diseases, 223 (76%) were caused by infections. | (6) Diseases of the digestive system: Digestive (n=131, 21%, 1% of affected athletes). | (3) Dermatologic system diseases: skin and subcutaneous tissue (n=53, 9%). |  |  | The highest incidence of injuries occurred in scooter cycling (38% of athletes injured), boxing (30%), mountain biking (24%), taekwondo (24%), water polo (19%) and rugby (19%). | The most common types of injuries were sprains/ligament ruptures (n=187), contusions/hematomas/abrasions (n=178), strains/muscle ruptures/tears (n=168), lacerations/abrasions/skin injuries (n=152), and tendinopathies/tendinopathies (n=112). | Common areas of injury were knees (n=130), thighs (n=108), ankles (n=103), face (n=94) and calves (n=90). |  |  |  |
| High precompetition injury rate dominates the injury profile at the Rio 2016 Summer Paralympic Games: a prospective cohort study of 51 198 athlete days | Web-based injury surveillance system. |  |  |  |  |  | Futsal (22.5), judo (15.5) and sevens (15.3) had the highest incidence of injuries. | Acute trauma is the most common injury in the Olympics. | Upper extremity injuries were the most common with an IR of 3.4 (95% CI 3.0 to 4.0), followed by lower extremity injuries with an IR of 3.0 (95% CI 2.6 to 3.5). |  |  | The 2016 Summer Paralympic Games in Rio saw the first athlete die during competition due to a head injury sustained during competition (cycling). This was clearly a catastrophic event and highlights the importance of ongoing efforts to plan for trauma and acute catastrophic events at major international multi-sport games. |
| Sport, sex and age increase risk of illness at the Rio 2016 Summer Paralympic Games: a prospective cohort study of 51 198 athlete days | Web-based Injury and Illness Surveillance System (WEB-IISS). | (7) Respiratory diseases: The respiratory system has the highest IR. | (3) Diseases of the skin system: skin and subcutaneous tissue. | (6) Diseases of the digestive system: digestive system. |  |  |  |  |  |  |  |  |
| Illness Among Paralympic Athletes: Epidemiology, Risk Markers, and Preventative Strategies |  | (7) Respiratory Disease: The IR for disease in Paralympic athletes is consistently highest in the respiratory system. | (3) Diseases of the skin system: skin and subcutaneous tissue. | (6) Digestive System Diseases: Gastrointestinal Tract (GIT). |  |  |  |  |  |  |  |  |
| Descriptive epidemiology of sports injury and illness during the Rio 2016 Olympic Games: a prospective cohort study for Korean team | Medical staff are required to record daily reports based on the IOC's Injury Surveillance System (ISS), which has been used at other Olympic Games since 2008 and is emailed to data management. | (7) Diseases of the respiratory system: Infections (39.7%, 29 diseases) were the most common cause of disease. Almost all infections occur in the respiratory tract (75.9%, 22 diseases). | (5) Diseases of the genitourinary system: Genitourinary infections were reported in 3 cases (10.3%). | (6) Digestive system diseases: gastrointestinal and skin infections were 2 cases (6.9%). |  |  | Boxing (600.0 injuries per 100 athletes), wrestling (500.0), taekwondo (380.0), handball (278.6), and fencing (235.7) had the highest incidence of injuries. |  | The four most frequent sites of injury for athletes were the ankle (13.0%, 40 injuries), lower back (11.7%, 36 injuries), knee (11.4%, 35 injuries), and thigh (9.7%, 30 injuries); 51.9% of these injuries occurred in the lower extremity and 24.0% in the upper extremity. |  |  |  |
| Incidence rate and burden of illness at the Pyeongchang 2018Paralympic Winter Games | Teams with their own medical support at the Paralympic Games use the Web-based Injury and Illness Surveillance System (WEB-IISS) to record illnesses (Online Supplementary Table 1), which has been used successfully at the London 2012 Paralympic Games, Sochi 2014 Paralympic Games and Rio 2016 Paralympic Games. | (7) Respiratory diseases: The highest number of diseases were reported in the respiratory system, with 28 diseases reported by 28 athletes (IR 4.1 [95% CI 2.9 to 5.9]). | (3) Dermatologic system disorders: The second most affected system was the skin and subcutaneous system, with 17 disorders reported by 16 athletes (IR 2.5 [95% CI 1.5 to 4.1]). Limb/prosthetic interface lesions, residual limb/skin subcutaneous pain, rashes, skin breakdown, and abscesses. | (14) Ophthalmology: The IR for the eye and ocular appendages (peripheral tissues) was 1.6 (95% CI 0.9 to 3.1), with 10 athletes reporting 11 diseases. Allergic conjunctivitis, corneal erosion, degenerative myopia, dry eye and ocular hypertension. |  |  |  |  |  |  |  |  |
| PyeongChang 2018 Winter Olympic Games and athletes' usage of a € polyclinic' medical services | Guiness analyzed electronic medical record data from athletes who visited the polyclinic between February 9, 2018 and February 25, 2018 with the Athlete Management Solutions (AMS) electronic medical record (EMR) system (GE Healthcare). | The most common of the respiratory diseases are upper respiratory infections (URIs), including sinusitis, rhinitis and acute pharyngitis and tonsillitis. | (16) Multiple systems: 107 cases in otolaryngology, including 3 cases of influenza. | (14) Ophthalmology: Ophthalmology. |  |  | The sport with the most injuries is alpine skiing, followed by snowboarding. |  | The most common site of injury was the knee (32%), followed by the spine, foot, hand and shoulder. |  |  |  |
| Experiences of the emergency department at the pyeongchang polyclinic during the 2010 pyeongchang winter olympic games | Medical records of all injured and sick patients at the PyeongChang Polyclinic were retrieved from the medical staff of the PyeongChang 2018 Winter Olympic Games Organizing Committee. Also, the same information during the Olympic Games was collected from data recorded by nurses at the polyclinic. | (7) Respiratory diseases: The respiratory system was the most commonly affected system in all categories: 45 cases among staff and 26 cases among athletes. The second most common symptom was respiratory symptoms (72 cases), including dyspnea, cough and sputum. Influenza tests were performed on 38 cases, of which 7 tested positive. | (6) Digestive disorders: The gastrointestinal tract was the second most commonly affected system of all categories: 28 cases in staff and 7 cases in athletes. Symptoms related to the gastrointestinal tract were the next most common, including diarrhea, vomiting, and gastrointestinal distress (37 cases). Rapid norovirus testing and polymerase chain reaction (PCR) were performed on 17 cases, of which four (all athletes) tested positive. |  |  |  |  |  | Analysis by injury site revealed that the most common injury sites among staff were fingers (14 cases) and hands (13 cases). Among athletes, the most common site of injury was the knee (8 cases), followed by the ankle (4 cases). |  |  |  |
| Winter adaptive sports participation, injuries, and equipment | The implementation of the International Paralympic Injury Surveillance System (ISS) has had a positive impact on the reporting of overall injury rates.ISS began reporting at the 2002 Winter Paralympic Games in Salt Lake City and found that 9.4% of athletes were injured. |  |  |  |  |  |  |  |  |  |  |  |
| Sports injury and illness incidence in the PyeongChang 2010 Olympic Winter Games: A prospective study of 2914 athletes from 92 countries | In this prospective cohort study, we used the IOC Integrated Sports Injury Surveillance System. We invited all National Olympic Committee (NOC) medical teams to report daily occurrences (or non-occurrences) of athlete injuries and illnesses using an electronic reporting form. At the same time, we retrieved the same information for all athletes treated for injuries and illnesses in the polyclinics operated by the PyeongChang 2018 Olympic and Paralympic Organizing Committee medical staff and all other medical sites. This data was collected through the Electronic Medical Record System (GE AMS). | (7) Diseases of the respiratory system: A total of 194 cases (70%) of diseases affected the respiratory system. Of the 194 respiratory diseases, 144 (74%) were caused by infections. | (6) Diseases of the digestive system: digestive system (n=36, 13%). | (3) Dermatologic system diseases: skin and subcutaneous tissue (n=15, 5%). |  | In preparation for and participation in competition, athletes and their support staff should be aware that several effective behavioral, nutritional, and training strategies exist to reduce exposure to pathogens and limit the degree of exercise-induced immunosuppression, thereby reducing the risk of disease. | The highest incidence of injuries occurred in ski u-tour (27.5 injuries per 100 athletes), snowboard cross-country (25.7 injuries per 100 athletes), ski cross-country (24.6 injuries per 100 athletes), snowboard slopestyle (10.1 to 32.3 injuries per 100 athletes), and ski aerials (2 to 6 injuries per 100 athletes); the lowest incidence of injuries occurred in Nordic biathlon, biathlon, snowboard slopestyle, snowboarding, and cross-country skiing (2 to 6 injuries per 100 athletes). The lowest incidence of injuries occurred in Nordic, biathlon, snowboard slopestyle, snowboarding and cross-country skiing (2 to 6 injuries per 100 athletes). | The most common types of injuries were sprains/ligament ruptures (n=62), bone contusions (n=57), and muscle contusions/hematomas (n=50). | The most common areas of injury were knees (n=53), ankles (n=34), hands/fingers (n=29) and lower back (n=27). |  |  |  |
| High incidence of injuries at the Pyeongchang 2018 Paralympic Winter Games: a prospective cohort study of 6804 athlete days | 1. The Web-based Injury and Injury Surveillance System (WEB-IISS) was used to record injuries to physicians who had their own medically supported teams at the Olympic Games;  2. The PyeongChang Local Organizing Committee customized the monitoring system at the medical polyclinic venues for use by teams without accompanying medical staff (n=8) as well as athletes who chose to use the polyclinic services covered by WEB-IISS. The system was used to record admissions to all surrounding hospitals, radiology services and all medical and pharmacy visits. It was also used to record specialty services at the central polyclinic and medical support at the Olympic venues. |  |  |  |  |  | Disability snowboarding has a higher injury rate (IR of 40.5) than all other sport categories combined. |  | Injury IRs were similar for the upper extremity (IR of 7.9) and lower extremity (IR of 7.1). The shoulder/upper arm/elbow complex had the highest injury IR (IR 5.7), followed by the head/neck/face complex (IR 4.3) and wrist/hand/finger complex (IR 2.2). | 1. Para snowboarding has relatively inexperienced participants compared to other established winter sports for the disabled, so it is recommended that education and safety programs be considered in this sport in the future;  2. In both summer and winter, the shoulder joint is a key concern for athletes with disabilities, primarily wheelchair users who use their upper body for sports and activities of daily living. Athletes use their arms during high-speed propulsion to gain strength and stability in their sport, thus suggesting that all sports with a high risk of shoulder injury require further investigation and subsequent intervention;  3. At the Sochi Olympic Games, research identified modifiable risk factors for alpine skiing injuries in people with disabilities, including (1) course design, (2) the number of training sessions allowed on the course and (3) the command and control structure between technicians and medical staff. At the PyeongChang Olympics, the Technical Committee implemented a number of changes to reduce the risk of injury, including: (1) redesign of the course, (2) an increase in the number of training sessions, (3) the ability to change the start time of the event and (4) the development of a snow contingency plan. All NPC medical and technical staff were also provided with educational opportunities to increase awareness and management of alpine skiing injuries for people with disabilities in this type of environment. |  |  |
| Close encounters of the US kind: Illness and injury among US athletes at the PyeongChang 2018 Winter Olympic Games | Team USA athletes were registered within the cloud-hosted EMR system (GE Centricity Software) prior to the competition. Pre-competition health history forms were sent electronically to each athlete by January 15, 2018 via a secure web-based patient portal. Report pre-participation health history responses to the Team USA sports medicine team via a secure web-based visual analytics dashboard (Tableau Server V.10.3.1; Tableau Software, Seattle, WA, USA) to identify and analyze significant findings and provide additional medical care or communication as necessary. | (7) Respiratory diseases: Respiratory (34) and are the most common diseases. | (3) Diseases of the dermatologic system: skin diseases. | (6) Digestive disorders: gastrointestinal tract (3). Eight gastrointestinal tests revealed a case of norovirus in a non-certified support staff member. |  | 1. Disease prevention efforts include evidence-supported hygiene kits that include hand sanitizers, mouthwashes, soaps, probiotic supplements, and jet lag prevention recommendations;  2. isolation of influenza patients from the WOC community to minimize the spread of influenza to WOC participants and staff. | The sports with the highest injury rates are ice hockey, alpine skiing, freestyle skiing and snowboarding. |  |  |  |  |  |
| Review of physiotherapy service for athletes of 2018 Olympic Winter games: consideration of preparation for two polyclinics | Medical records, visit forms, and data documenting all physical therapy visits were coded into a customized electronic medical record (EMR) system provided by GE. All data was entered into the IOC eMedical Services Physical Therapy Services form provided by the IOC. |  |  |  |  |  | The top three sports with injury rates of more than 50 per 1,000 athletes are alpine skiing, cross-country and snowboarding on mountain venues, and figure skating, curling and speed skating on urban venues. |  | Lumbar spine, knee and multiple body injuries. The most treated sites at the District Polyclinic were lumbar spine, knee and multiple sites (24.1%, 14.9% and 12.3% respectively). The most treated sites at the District Polyclinic were thigh, neck/neck, and hip/pelvis/sacrum/gluteal (13.1%, 10.5%, and 10.5%, respectively). The prevalence of lumbar spine injuries was significantly higher in the mountain polyclinics than in the urban polyclinics (24.1% in the mountain areas versus 7.8% in the urban areas). This reflects a higher incidence of lumbar spine injuries in mountain polyclinics than in urban polyclinics. | Athletes seeking physical therapy services at the Winter Olympics are highly susceptible to overuse injuries and should therefore be aware of injury prevention strategies for two different venues for Winter Olympic sports (i.e., Mountain Winter Olympic sports and Urban Winter Olympic sports). |  |  |
| Surveillance To Prevent The Spread Of Norovirus Outbreak From Asymptomatic Food Handlers During The PyeongChang 2018 Olympics |  | (6) Digestive disorders: 5 out of 707 samples were identified as norovirus. kim et al. reported that in the Olympic Village, only 4 patients were diagnosed with NoV and all were athletes. |  |  |  |  |  |  |  |  |  |  |
| Incidence and burden of illness at the Tokyo 2020 Paralympic Games held during the COVID-19 pandemic: a prospective cohort study of 66 045 athlete days | WEB-IISS was used by 3836 (87%) athletes from 81 countries (50%) and 567 (13%) athletes from 81 countries used the polyclinic and venue facilities. Athletes were prospectively monitored for 15 days, including before and during the Tokyo 2020 Paralympic Games. | (3) Diseases of the skin system: the skin system. | (7) Respiratory diseases: respiratory system. | (6) Diseases of the digestive system: gastrointestinal system. | (18) COVID-19 (SARS-CoV-2): 13 cases of respiratory disease were diagnosed as COVID-19 (0.2 (95% CI 0.11 to 0.34)). Five of these cases occurred in the sport of judo and four cases were from the same country. Two cases were reported in swimming and athletics. | 1. Effectiveness of the strict COVID-19 response implemented during the Tokyo Paralympic Games. Some of the strategies employed included mandatory vaccination and mask use, thorough hygiene, early recognition of COVID-19 symptoms, use of the "test-trace-segregate" principle (i.e., testing for COVID-19 on a daily basis and segregation of athletes who tested positive), and restriction of movement of all participants in the Olympic environment.  2. These measures include heat panels with full-body cooling options in medical support areas of competition venues, preparatory heat acclimatization for teams, measurement of wet bulb black globe temperatures (WBGT) at each venue, and scheduling of competitions on cooler mornings and evenings;  3. pay attention to skin conditions. For example, physicians should be aware of skin stress and changes in temperature and humidity during long trips;  4. countermeasures such as thorough hand hygiene and reduced human contact may help reduce gastrointestinal and genitourinary morbidity. |  |  |  |  |  |  |
| Sports Injury and Physiotherapy Services in the 2018 PyeongChang Winter Paralympic Games: Considerations and Potential Recommendations for Future Paralympics | A total of 201 participants (51 athletes and 150 non-athletes) were admitted from March 1, 2018 to March 20, 2018 and utilized IPC physical therapy services. Physical therapists used the IPC EMS Physical Therapy Services Form to document the initial examination of admitted patients. The Electronic Medical Record (EMR) is designed to provide basic demographic information, basis of injury, physical assessment, and a list of available physical therapy treatments. A clear picture of the injury (i.e., acute, recurrent, or chronic) is needed to better understand and determine the treatment and exercise therapy discipline to be utilized. |  |  |  |  |  |  | The most common type of injury for both athletes and non-athletes was muscle strains/tears (n = 95, 47.3%). | The most commonly injured anatomical sites in athletes were the shoulder and spine (n = 14), followed by the head/neck (n = 7). Among non-athlete clients, the spine was the most commonly injured site (n = 55), followed by the shoulder (n = 39) and head/neck (n = 21). | 1. Soft tissue injuries are the most common type of injury among athletes with disabilities. Our findings emphasize the importance of pre-training warm-up and physical training. |  |  |
| Medical services for sports injuries and illnesses in the Beijing 2022 Olympic Winter Games | Information was collected on all Athletes treated for injuries and illnesses at the Polyclinics and all other medical sites operated by the Beijing 2022 Medical Staff between February 4 and 20, 2022, as well as ambulance transfers. These data were collected using an electronic medical record system. | (15) Dental: 28 cases. | (16) Multiple systems: 22 cases in ophthalmology and ENT. | (17) Unknown or not specified: The most common cause of illness was other (past medical history, medications) (n=52, 65%). Other included other genitourinary, gynecological, cardiovascular, neurological, psychiatric, musculoskeletal. |  | 1. In order to prevent the spread of C pneumonia during the Olympic Games, the organizers of the Beijing 2022 Winter Olympic Games have developed scientific and pragmatic response measures. In particular, they adopted an innovative "closed-loop" system [29]; categorized participants and competition venues according to high, medium and low risk levels, and developed 12 strict response measures. These measures included: a) participants were required to complete vaccinations at least 14 days prior to departure for China; and b) participants who had not completed vaccinations were required to be quarantined for 21 days upon arrival in Beijing. As a result, the incidence of respiratory illnesses, including respiratory infections, decreased significantly. | The sport with the most injured athletes was Ice Skating (104), followed by Alpine Skiing (53), Ice Rink (37), Freestyle Skiing (36), Ice Hockey (35), with the least number of injuries occurring in the Nordic Skiing program (20), and no athletes were injured in Curling. | Muscle strains (29.1%), impingement (16.6%) and contusions (12.6%) were the most common types of injuries. | The knee (22.1%) was the most injured site, followed by the lumbar spine (10.4%) and thigh (9.2%). |  |  |  |
| Incidence Of Injuries Among Team USA Athletes Competing In Tokyo 2020 Summer Olympic Games |  | (1) Certain infectious or parasitic diseases: the most common disease is infection (23.5%). | (6) Diseases of the digestive system: gastrointestinal diseases (15.7%). | (3) Diseases of the skin system: skin diseases (13.7%). |  |  |  |  |  |  |  |  |
| New sports, COVID-19 and the heat: Sports injuries and illnesses in the Tokyo 2020 Summer Olympics | All National Olympic Committee (NOC) medical teams were invited to report daily occurrences (or lack thereof) of athlete injuries and illnesses using the electronic reporting form. At the same time, the same information was retrieved for all athletes treated for injuries and illnesses at the polyclinics operated by the Tokyo Organizing Committee for the Olympic and Paralympic Games (Tokyo 2020/OCOG) medical staff and at all other medical sites. These data were collected through the Electronic Medical Record System (GE AMS). | (3) Diseases of the skin system: dermatologic system (n=83, 19%). | (11) Heat-related illness: thermoregulatory system (n=78, 18%). All were exercise-induced heat stroke. | (7) Respiratory diseases: Respiratory system (n=75, 17%). Of the 75 respiratory illnesses, 45 were caused by infections (10% of all illnesses, 0.4% of athletes developed respiratory infections), of which 18 were SARS-CoV-2 infections (4% of all illnesses, 0.16% of athletes). | 18 cases were SARS-CoV-2 infections (4% of all illnesses and 0.16% of athletes). In addition to the cases that occurred during the study period (July 23-August 8, 2021), 15 confirmed cases occurred before the start of the Olympic Games (i.e., from the time of arrival at the airport to the Opening Ceremony of the Olympic Games), for a total of 33 COVID-19 cases in athletes (0.29% of athletes). | 1. Key public health principles included mandatory wearing of face masks; minimization of physical contact; testing, tracing and isolation; and increased personal hygiene (hand washing). All participating NOC athletes were also vaccinated prior to the Games, and the vaccination rate for Olympic Village residents was 85%. For testing purposes, participants were required to provide proof of two negative PCR tests prior to departure for Tokyo, another negative test upon arrival, and then daily screening tests throughout the Games. A significant decrease in respiratory infections (0.4% of athletes developed upper respiratory infections), compared to previous Olympic Games (4.8% at PyeongChang, 1.9% at Rio, 4.2% at Sochi, 1.9% at London, 1.1% at Vancouver); and  2. Heat acclimatization educational materials - one of the most effective measures to protect athletes' health and performance prior to competing in hot environments - have been developed and widely distributed to athletes to help them prepare for the Games. Training in the heat for approximately 2 weeks triggers physiological adaptations that lead to better thermoregulation, reduced cardiovascular stress and improved exercise capacity in the heat. Prior to the Games, two events (marathon and racewalking) were moved 800 kilometers north to Sapporo, where temperatures are typically 4-5°C cooler than in Tokyo; however, during the Games it was just as hot and humid. Other events were rescheduled for cooler mornings or evenings (e.g. women's soccer finals). A number of important venue cooling strategies were also implemented before, during and after the games, including the provision of hydration, shading, air conditioning, fans, ice packs, misting, and pre-cooling and recovery ice baths. In terms of medical management, specific medical algorithms have been developed for high-heat stress venues with the aim of early identification and diagnosis, rapid on-site cooling and advanced clinical care. | The highest incidence of injuries was in boxing, BMX racing, BMX freestyle, skateboarding, karate and handball, while diving, road cycling, rowing, marathon swimming and shooting had the lowest incidence of injuries. | The most common types of injuries were skin lacerations/damages/abrasions (n=133), grade 1 or 2 muscle strains (n=119), and grade 1 or 2 ligament sprains (n=113). | The most common areas of injury were the knee (n=114), the back of the thigh (n=89), the shoulder (n=88), the ankle (n=86), and the face (including eyes, ears, and nose; n=80). |  |  |  |
| Lessons from the Winter Paralympic Games disclosing the epidemiology of winter sports injury in paralytic athletes: a meta-analysis |  |  |  |  |  |  | Alpine skiing, Nordic skiing, and sled field hockey are the three main sports with more reports of associated sports injuries. |  |  |  |  |  |
| Emergency department activities at the Athletes' Village during the Tokyo 2020 Olympic and Paralympic Games | Data were collected using an electronic medical record system, nursing records, and questionnaires administered during the July 13-September 8, 2021 triage period. Comprehensive clinic data involving certified athletes and team members was summarized. | Olympic: (8) Musculoskeletal disorders: Musculoskeletal problems (26 cases);  Paralympics: (16) Multiple systems: ear, nose and throat problems were the most common (21 cases). | Olympic: (6) Digestive system disorders: gastrointestinal disorders, including abdominal pain (13 cases);  Paralympics: (8) Diseases of the musculoskeletal system: musculoskeletal problems (19 cases). |  |  | Heat acclimatization educational materials were prepared and widely distributed to athletes to help them prepare for the Games. It was also anticipated that patients suffering from heat stroke would visit the polyclinics. As a result, a variety of measures were taken to prevent heatstroke, including fluid supplementation, frozen intravenous fluids, air conditioning, fans, ice packs, misting and ice baths. |  |  |  |  |  |  |
| Incidence of injury and illness among paediatric Team USA athletes competing in the 2020 Tokyo and 2022 Beijing Olympic and Paralympic Games | The electronic medical records system records all injuries that occur during competition during the four Olympic Games. | (1) Certain infectious or parasitic diseases: infections. | (6) Diseases of the digestive system: diseases of the gastrointestinal tract. |  |  |  |  |  | The limb is the anatomical site with the highest percentage and incidence of injury. More specifically, the ankle and knee are the most commonly injured anatomical sites. | 1. Healthcare providers working with elite female pediatric athletes should focus on preventatively reducing the risk of head injuries, lower extremity injuries, and progressive injuries and use techniques previously determined to be effective;  2. the care of pediatric athletes, both elite and non-elite, requires special consideration in terms of mental health, hormonal maturation, and injury characteristics. |  |  |
| Acute in-competition medical care at the Tokyo 2020 Olympics: A retrospective analysis | (1) daily reported data from medical operations managers (number of cases reported by medical operations managers at each venue)10; (2) Japanese version of the Post Extreme Emergency and Disaster Surveillance (abbreviated as J-SPEED) data (non-athlete-reported data); (3) electronic medical registry (EMR) data (records of athlete treatments); (4) treatment data from the hospitals; (5) main operations center data (information from medical coordination headquarters) and (6) final diagnosis information from treating hospitals. | (11) Heat-related illnesses: A total of 225 cases of heat stroke occurred during the Games, of which 100 were treated in the athletes' clinic and 125 in the spectators' clinic. The highest incidence of heat-related illnesses occurred in track and field (marathon and race walk; 136), followed by freestyle swimming on small wheels (56) and marathon swimming (39). | Athletes Clinic: (14) Ophthalmology: vision problems;  Spectator Clinic: (17) Unknown or not specified. | Athlete Clinic: (17) Unknown or not specified;  Spectator Clinic: (10) Neurological Disorders. |  | 1. As a result, the start times of some events (tennis, golf, soccer and women's marathon) were changed to times of cooler temperatures, such as early morning or late evening. In addition to sufficient quantities of oral rehydration drinks, the Athletes' Clinics at the 32 venues were equipped with ice towels, athletes' lounges, portable coolers, fans, mist fans and ice baths. As a precautionary measure, WBGT-based notifications were sent not only to athletes' smartphones, but also to staff members who wore masks to control infections in hot environments;  2. Various heat stroke countermeasures were effective, especially the creation of a heat stroke deck. For athletes and officials, changing the time of competition, such as holding high-risk events early in the morning or after sunset, and rapid on-site cooling by the venue medical staff immediately after the onset of the illness were particularly effective. As a result, emergency off-site transportation of athletes was significantly reduced. We also believe that the reduction in footfall due to non-spectator events, the cancellation of live broadcasts, the digital distribution of live cheering, the requirement at many venues to stay at home during non-spectator events, and the effectiveness of transportation demand management also contributed to the reduction in heatstroke as a preventive measure;  3. several measures were taken in response to COVID-19: standard precautions, especially wearing masks and observing hygiene; special border protection procedures, such as airport arrival restrictions; and two PCR tests prior to entry;  4. the cooperation of emergency physicians, including the management of diseases such as heat stroke, and sports physicians, including the management of injuries. | The highest incidence of injury was in golf 70.8, followed by boxing 13.8, rock climbing 12.5 and skateboarding 11.3. |  |  |  |  |  |
| Characteristics of Sports Injuries in Athletes During the Winter Olympics: A Systematic Review and Meta-analysis |  |  |  |  |  |  | Snow and ice sports had the highest incidence of injuries (11.3%), with the top 3 sports being snowboard cross-country (31.4%), freestyle aerials (28.6%), and snowboard slopestyle (27.7%). | The most common types of injuries are contusions/hematomas/contusions, sprains (dislocations, subluxations, instabilities, ligaments, ruptures) and strains (muscle ruptures, tears, tendon ruptures). | The most common areas of injury are the knee, head and ankle. | We hope that the results of this study will help provide the data needed for the development, application, and evaluation of injury causation and prevention models. The International Olympic Committee (IOC), National Olympic Committees (NOCs), and International Federations (IFs) should improve the monitoring and protection of athletes to reduce the incidence of injuries. For example, research into the mechanisms of injury and the precise biomechanical factors involved in movement techniques in major competitions can help provide evidence-based injury prevention measures for every athlete. Enhance the comprehensive capacity of medical services, improve the professionalism of medical staff, intervene in early rehabilitation, promote mental health, and improve the level of service. Future research should be combined with virtual simulation technology to explore whether the inclusion of virtual simulation training can reduce the incidence of injury in difficult maneuvers. |  |  |
| Exploring the Epidemiology of Injuries in Athletes of the Olympic Winter Games: A Systematic Review and Meta-Analysis |  |  |  |  |  |  | The sports with the highest injury rates are freestyle skiing, snowboarding, alpine skiing, bobsledding and ice hockey. | Throughout the Winter Olympics, the most common types of injuries were contusions, hematomas and abrasions, which accounted for 17.2 percent of all injury categories. This was closely followed by strains, including muscle ruptures, tears and tendon ruptures (17.1%), and sprains, including dislocations, subluxations and ligament ruptures (15.4%). | Injuries to athletes are concentrated in specific areas of the body, with the knee being the most susceptible, accounting for 16.5% of all recorded injuries. This was closely followed by chest/lumbar/back and wrist/hand/finger injuries at 10.5% and 10.3% respectively. | 1. There are significant differences in sports injuries across sports, venues and genders, and these differences need to be taken into account in the provision of medical aid services to athletes. Particularly in snow sports and ice hockey, where the risk of injury is higher, adequate medical equipment and specialized medical personnel must be prepared. For ice hockey, attention should be paid to common injuries such as contusions, sprains, muscle ruptures, tendon ruptures and fractures. For alpine skiing and snowboarding events, attention should be paid to common injuries such as dislocations, ligament ruptures and fractures. In addition, considering the diversity of athletes participating in the Winter Olympics, the allocation of male and female medical staff should be planned according to the unique injury characteristics of different sports and genders;  2. Emphasize the professional training of medical personnel, both in terms of first aid instruction and knowledge of the rules of the games and the characteristics of the events, to ensure that sports injuries occurring during the games are treated in a timely and effective manner. The training should focus on the types of injuries commonly seen in the Winter Olympics and emergency treatment methods, including contusions, muscle breaks and sprains on the knees, thoracic spine, lumbar spine, back, hands, ankles, thighs, head, shoulders and collarbones. In sports injuries, the timeliness of first aid measures is crucial, and medical personnel must have the ability to diagnose and treat quickly;  3. Enhancing protective measures at competition venues is imperative. The design of protective facilities for snowmobile tracks and freestyle skiing courses should be optimized to enhance athlete safety and minimize potential injury risks. This includes the use of more shock-absorbing materials, ensuring that guardrails are sufficiently high, and paying special attention to the placement of end-of-course protective structures to prevent athletes from colliding with dangerous objects. |  |  |
| Injury incidence according to athlete impairment type during the 2012 and 2016 Summer Paralympic Games: a combined analysis of 101 108 athlete days |  |  |  |  |  |  | The sports with the highest prevalence were futsal (FB5) (25.9), wheelchair fencing (WF) (17.06) and judo (15.5). |  | upper limb |  |  |  |
| Team USA injury and illness incidence at the 2022 Beijing Winter Olympic and Paralympic Games | Injuries and illnesses were recorded for the Olympic Games from January 24 to February 24, 2022 (pre-event: January 24 to February 3, competition: February 4 to February 20, post-event: February 21 to February 24) and for the Paralympic Games from February 21 to March 16, 2022 (pre-event: February 21 to March 3, competition: March 4 to March 13, post-event: March 14 to March 16) Post-Competition: March 14 to March 16). The U.S. Olympic and Paralympic Committees' Injury and Illness Surveillance Program during the 2022 Beijing Olympic and Paralympic Games was used. | Olympic: (1) Certain infectious or parasitic diseases: The most frequent diseases among athletes are infectious diseases;  Paralympics: (7) Respiratory diseases: the most common diseases among athletes are respiratory diseases. |  |  |  |  | Among Olympic sports, bobsledding (IR = 51.5 injuries/1000 athlete-days), skiing and snowboarding (IR = 22.6 injuries/1000 athlete-days), and bobsledding and snowmobiling (IR = 17.7 injuries/1000 athlete-days) had the highest injury rates. |  |  |  |  |  |
| Incidence of injury and illness at the Beijing 2022 Paralympic Winter Games held in a closed-loop environment: A prospective cohort study of 7332 athletes A prospective cohort study of 7332 athlete | Injuries and illnesses of all teams are recorded daily by the team medical staff through web forms, local organizing committee medical (polyclinic) facilities and venue medical support. | (7) Respiratory diseases: respiratory system. | (6) Digestive diseases gastrointestinal system. | (3) Diseases of the skin system: the skin system. | Three cases of respiratory disease were diagnosed as COVID-19. All three cases occurred in the sport of Nordic skiing (biathlon) for people with disabilities, and two of them were from the same country. | The low respiratory morbidity may be attributed to the impact of the strict COVID-19 response implemented during the Beijing Olympics, including, but not limited to, minimal physical contact, wearing masks, frequent hand washing, and use of hand sanitizers. | Alpine skiing has a significantly higher injury rate than ice hockey, Nordic skiing and wheelchair curling. (In addition, snowboarding has a significantly higher injury rate than Nordic skiing). |  | The shoulder/upper arm/elbow complex had the highest incidence of injury, followed by the head/neck/face complex. |  |  |  |
| The Tokyo 2020 and Beijing 2022 Olympic Games held during the COVID-19 pandemic: planning, outcomes, and lessons learnt |  |  |  |  | During the Tokyo Olympics, a total of 464 people tested positive, with an overall positivity rate of less than 0.069%. All positive results were confirmed by PCR. During the Olympic Games, 33 athletes and 34 officials accompanying the team tested positive. For the Beijing Winter Olympics, 437 people were confirmed positive, with an overall positive rate of 0-023%. During the Olympic Games, 98 athletes and 82 accompanying officials tested positive. | 1 Following the decision to postpone the Tokyo Games, an organizational infrastructure was established to ensure effective collaboration and cross-sectoral work to plan for the rescheduled Games. At the heart of this infrastructure was the All-Partner Working Group, which included senior representatives from the Japanese government, the organizing committee, and the IOC, and was charged with developing and overseeing a core plan for planning the 2021 Summer Olympics. In terms of public health, this planning includes the formation of a multi-sectoral independent expert panel, taking into account the recommendations of the Japanese public health system and the WHO. The All-Partner Working Group prioritized evidence-based decision-making in developing overall guidelines for the Games. Preparation work focused on key WHO messages on COVID-19 and measures that communities should take to reduce the risk of SARS-CoV-2 transmission; 2. The work examined scenario planning to explore what the epidemiology of COVID-19 in Japan and globally might look like in the summer of 2021; and identified and prioritized countermeasures most likely to make the greatest difference in reducing the risk of COVID-19 during the Games. 19 risk; countermeasures (including screening and case management) that are most likely to make the greatest difference; the potential for new treatments and vaccines to change the risk profile; and risk communication to ensure a clear, shared understanding of risk and countermeasures;  3. recommends reducing the risk of COVID-19 transmission by relying on good disease surveillance, implementation of good public health and social measures, and effective detection, tracing, and isolation systems. High vaccination rates and good risk communication to support high adherence will add another layer of protection. |  |  |  |  |  |  |
| Injuries and illness of athletes at the Tokyo 2020 Olympic and Paralympic summer games visiting outside facilities | Records provided by the Organizing Committee's Medical Department were categorized to obtain the following information: 1) Medical Operations Manager Daily Report data (the number of cases reported by the Medical Operations Manager at each venue); 2) Japanese version of Extreme Emergency and Post-Disaster Surveillance (referred to as J-SPEED) data (non-athlete report data); 3) Electronic Medical Registration (EMR) data (records of athletes' treatments); 4) External Medical Facility Attendance data; 5) Main Operation Center data (information provided by the Medical Coordination Headquarters); and 6) Final diagnosis information from the treating hospital. | Olympic: (17) Unknown or not specified: other diseases (n = 13);  Paralympics: (7) Respiratory disease: respiratory system. | Olympic: (16) Multiple systems: ENT disorders n = 5, 38.5%);  Paralympics: (3) Diseases of the dermatologic system: diseases of the dermatologic system. | Olympic Games: (11) Heat-related illnesses: 5 cases;  Paralympics: (6) Digestive system diseases: digestive system cases. | With the exception of coronavirus disease (COVID-19), there was only one case of an infectious disease (malaria infection) that led to a patient's visit to an external health facility. | 1. In the future, external facilities supporting the Convention should establish hospitals capable of dealing with general emergencies and in the field of otorhinolaryngology;  2. Responding to heat-related illnesses: this includes the establishment of an initial screening system and the provision of oral rehydration salts at each venue. Considering the high temperatures in the Tokyo metropolitan area, the fact that the marathon and race walk were held in the northern Japanese city of Sapporo was also affected. | Olympic Games: soccer had the highest incidence (n = 16), followed by track cycling (n = 5) and judo (n = 4);  The sport with the highest frequency of injuries was cycling road racing (n = 4), followed by weightlifting (n = 2) and track and field (n = 1). |  | At the Olympics, the most common injuries (n = 48) were extremity injuries (n = 25, 52.1%), followed by head injuries (n = 11, 22.9%), facial injuries (n = 6, 12.5%), cervical spine injuries (n = 3, 6.3%), and chest injuries (n = 3, 6.3%). The most common Paralympic injuries (n = 7) were extremity injuries (n = 4, 57.1%), with 2 (28.6%) head injuries and 1 (14.3%) chest injury. |  |  |  |
| Olympic Games during nationwide lockdown: Sports injuries and illnesses, including COVID-19, at the Beijing 2022 Winter Olympics | The number of injuries and illnesses suffered by athletes on a daily basis was documented through (1) reports from all National Olympic Committee (NOC) medical teams and (2) reports from Beijing 2022 medical staff at polyclinics and medical sites. | (7) Respiratory diseases: Respiratory system (n=52, 48%). Of the 52 respiratory illnesses, 41 were caused by infections (28% of all illnesses, 1.4% of athletes developed respiratory infections), 32 of which were SARS-CoV-2 infections (29% of all illnesses, 1.1% of athletes). | (6) Digestive disorders: gastrointestinal (n=14, 13%). | (3) Diseases of the dermatologic system: skin diseases (n=9, 8%). | 32 cases were SARS-CoV-2 infections (29% of all diseases, 1.1% of athletes). In addition to the cases that occurred during the study period (February 4, 2022 to February 20, 2022), 66 confirmed cases occurred before the start of the Olympic Games (i.e., from the time of arrival at the airport to the Opening Ceremony of the Olympic Games), with a total of 98 cases of COVID-19 in athletes (3.4% of athletes). | 1. Key measures included a vaccination policy; testing, tracking and quarantine; dedicated transportation arrangements, with no use of public transportation; social distancing in villages and venues; good ventilation in all indoor areas; wearing of masks; and a focus on personal hygiene (hand-washing). In terms of testing, participants were required to provide proof of two negative PCR tests before departing for Beijing, another negative test on arrival, and then daily screening tests throughout the Games. However, during the Olympic Games, China implemented a national zero COVID policy, which led to the implementation of some important additional stringent countermeasures;  2. A "closed-loop" management system minimized the potential for SARS-CoV-2 transmission in China by ensuring almost complete isolation of international participants and the local workforce inside the ring from the general population outside the ring. In addition, the vaccination policy was more stringent, as vaccination was mandatory (recommended in Tokyo), pre-arrival testing was more comprehensive, requiring PCR testing throughout (antigen testing was performed at Tokyo airports), isolation of anyone testing positive for SARSCoV-2 was longer (14 days or until consecutive daily negative tests were performed, whichever was shorter), and all close contacts were All close contacts were also quarantined for a longer period of time. | The highest injury rates were recorded in ski u-turn (30.2 injuries per 100 athletes), ski jump (28.1 injuries per 100 athletes), snowboard slopestyle (23.3 injuries per 100 athletes) and ski slopestyle (between 10.2 and 34.6 injuries per 100 athletes), while the lowest rates of injury were recorded in curling, alpine mixed team parallel steeplechase, Nordic and alpine super slalom (between 0.9 and 2.2 injuries per 100 athletes). The lowest incidence of injuries was recorded in curling, alpine mixed team parallel slalom, Nordic biathlon and alpine giant slalom (0.9 to 2.2 injuries per 100 athletes). | The most common types of injuries were joint sprains/ligament tears (n=62), muscle strains/ruptures/tears (n=33), superficial contusions/abrasions (n=29), and muscle contusions (n=23). | The most common areas of injury were knees (n=38), head (n=35), shoulders (n=32), lumbosacral spine/hips (n=30), and hands (n=23). | Although injury risk factor and mechanistic studies are needed to determine the etiology of each sport and discipline, longitudinal surveillance of injuries and diseases is important to detect changes in injury incidence and characteristics over time. |  |  |
| Management and nursing practice for the patients in the polyclinic of Beijing Olympics Village | The clinic reports the number of patients and basic information that occurred between the previous day and the current day by the end of each day, and transmits them to the IOC through a dedicated line. This set of patient data is uploaded to the IOC and includes basic information such as name, gender, age, diagnosis, country, and identity of patients treated at the Olympic Village Polyclinic on a daily basis, as well as statistics on respiratory illnesses, gastrointestinal illnesses, injuries, and other illnesses in accordance with the criteria for classifying illnesses established by the IOC Athletes' Commission. | (7) Diseases of the respiratory system: Diseases of the respiratory system, a total of 855 cases accounting for 10.21%. | (14) Ophthalmology: 736 cases of eye diseases accounting for 8.79%. | (16) Multiple systems: 596 cases of ENT diseases accounted for 7.12%. |  |  |  |  |  |  |  |  |
| [Sports Injuries and Illnesses of the German National Team during the 2016 Olympic Summer Games in Rio de Janeiro] |  | (7) Diseases of the respiratory system: a total of 164 cases were treated for upper respiratory tract infections. |  |  |  |  |  |  |  |  |  |  |
| Beyond London 2012: The quest for a security legacy |  |  |  |  |  |  |  |  |  |  |  |  |
| Upcoming Paralympic summer games in Rio: What did the German medical team learn from the London Games? | Using a standardized visit form, all sports-related musculoskeletal conditions (MSCs) are documented, as well as medical care for accompanying personnel, whether or not time is lost due to training or competition. |  |  |  |  |  | The incidence of musculoskeletal disorders in German athletes was 62.9±15.4/1000 athlete-days. | The majority of injuries occurred during training (87%) rather than during competition (6%) or leisure time (7%). While the majority of athletes were able to continue their sporting activities (77%), performance ability was limited in 43 cases (20%) and 4 athletes (3%) were unable to continue. | The most common diagnoses were myalgia (N.=105), tendinopathy (N.=14), and unspecified back pain (N.=14). |  |  |  |
| Common cold in Team Finland during 2018 Winter Olympic Games (PyeongChang): Epidemiology, diagnosis including molecular point-of-care testing (POCT) and treatment Epidemiology, diagnosis including molecular point-of-care testing (POCT) and treatment | Team members participated in surveillance throughout the Olympic trip. All team members were instructed to immediately report symptoms of upper respiratory infection to the chief medical officer (MV) who lived in the same building. At the onset of symptoms, two samples of nasal mucus (one from each nostril) were collected at a depth of 3-4 centimeters using a flocked nasal swab. One sample is used immediately for POCT and the other is refrigerated in a dry storage tube in the team medical room. Swabs were also collected from subjects who had been exposed to patients with colds. | (7) Respiratory diseases: In the field, 11 out of 42 (26%) symptomatic cases were diagnosed with respiratory causes. Prior to the match, two cases of Influenza B were detected by antigenic testing at local medical facilities. POCT in the team medical room detected one case of Influenza A virus, three cases of Influenza B virus and five cases of Respiratory Syncytial Virus A. Six athletes and five staff members were diagnosed with a common cold etiology. |  |  |  | 1. Strict hand-washing, use of hand sanitizers, increased disinfection and strict isolation of patients may prevent the spread of the virus within the team, but are clearly ineffective. This is due to the multifactorial mechanism of transmission of viral infection;  2. In addition to early treatment with oseltamivir, POCT can be effective for post-exposure oseltamivir prophylaxis in close contacts (e.g., people living in the same residence or traveling on the same flight or in the same vehicle);  3. isolation of infected team members begins after the onset of symptoms and continues for 3-4 days (i.e. the most infectious period). |  |  |  |  |  |  |
| Are rapid antigen SARS-Cov-2 tests effective for mass screening of travelers at airports? The Olympic experience | Japanese health authorities have instructed quarantine stations to replace the nasopharyngeal swab (NPS) RT-qPCR or LAMP technique with the Lumipulse® SARS-CoV-2 antigen assay to screen asymptomatic carrier passengers at international airports using saliva- or NPS-based samples. |  |  |  | (18) COVID-19 (SARS-CoV-2): Since 1 July, the Tokyo Organizing Committee for the Olympic Games (TOCOG) has recorded 430 cases of Olympic-related infections. Nearly 624,000 screening tests were carried out and the infection rate was 0.02%. Of these positive patients, only 32 stayed in the Olympic Village, 29 of whom were athletes. The largest number were contract workers, followed by Olympic participants (236 and 109, respectively). | 1. The case of a Ugandan Olympic athlete who tested negative on entry and was allowed to travel by bus to the host city in Japan, but developed symptoms shortly thereafter, caused an outcry in the media and led to a tightening of screening at the airport. The driver and several city officials had to be quarantined. This incident highlighted the inherent limitations of detection methods based solely on the detection of SARS-Cov-2 antigen;  2. The organizers created an Olympic "bubble" - a set of venues, hotels and media centers - within which most of the participants in the Games were confined;  3. A combination of factors, including a vaccination rate of over 70% among Olympic athletes and other participants, extensive routine testing, social distancing, and a ban on domestic and international spectators, undoubtedly played a key role in the low rates of infection observed;  4. additional strategies such as supervised isolation, frequent re-testing and close follow-up of positive patients were carefully implemented. |  |  |  |  |  |  |
| COVID-19 infection during the Olympic and Paralympic Games Tokyo 2020 | Athletes and officials are tested daily for quantitative salivary antigens and for salivary real-time polymerase chain reaction (RT-PCR) following a positive antigen test. Other personnel undergo periodic salivary RT-PCR testing with varying frequency depending on the level of contact with the athlete. |  |  |  | (18) COVID-19 (SARS-CoV-2): Between June 29, 2021 and September 8, 2021, more than one million tests were performed on participants, and SARS-CoV-2 infection was confirmed in 41 OGT/PGT athletes and 822 OGT/PGT non-athletes. The estimated incidence rate was 0.24% for OGT athletes and 0.30% for PGT athletes. Of the 41 athletes, 17 cases had no detailed information, only one clustered case was identified in 5 Greek OGT synchronized swimmers, and 19 cases were scattered across sports. All were non-Japanese and 40 positive cases (97.6%) were detected during the 14-day isolation period after arrival, with only one Italian rower diagnosed after isolation. Of those infected with SARS-CoV-2 in OGT and PGT, 68.2% and 74.4% were Japanese. Therefore, approximately 0.34% of foreign OGT non-athletes and 0.56% of foreign PGT non-athletes were infected during the study period. | The two core infection control strategies used in tournaments are bubble programs and frequent testing.  1. The Bubble Plan consists of a series of measures to isolate participants from the public. Athletes are not allowed to leave their accommodations or the competition venue;  2. they may only use the transportation system prepared for these events and may only contact a pre-submitted list of individuals;  3. In terms of testing strategies, athletes and officials undergo daily quantitative saliva antigen testing and salivary real-time polymerase chain reaction (RT-PCR) testing following a positive antigen test. Other personnel receive regular salivary RT-PCR testing with varying frequency depending on the level of contact with the Athlete;  4. Athletes and persons from abroad must also undergo two COVID-19 tests, such as RT-PCR and quantitative antigen testing, within 96 hours prior to flight departure, quantitative saliva antigen testing upon arrival, and quarantine and daily quantitative saliva antigen or saliva RT-PCR testing for the first three days;  5. encourage vaccination, prohibit spectators from entering and require all to wear masks. In terms of vaccinations, over 80% of athletes and staff were vaccinated, but vaccinations were not mandatory. |  |  |  |  |  |  |
| SARS-CoV-2 Infections in Close Contacts of Positive Cases in the Olympic and Paralympic Village at the 2021 Tokyo Olympic and Paralympic Games | 1. All participants (e.g. athletes, coaches, medical staff, physiotherapists and Paralympic escorts) were required to undergo SARS-CoV-2 testing prior to departure from their country of origin; all were tested for SARS-CoV-2 salivary antigens upon arrival at Japanese airports; and all participants underwent laboratory salivary antigen screening each morning. Those who screened positive underwent confirmatory nasopharyngeal polymerase chain reaction (PCR) testing;  2. Close contact data for participants who tested positive were collected from July 13, 2021 to August 11, 2021 for the Olympic Games and from August 17, 2021 to September 8, 2021 for the Paralympic Games at the Close Contact Testing Area in the Olympic Village (hereafter referred to as the "Area"). |  |  |  | COVID-19 (SARS-CoV-2) (18) COVID-19 (SARS-CoV-2): At the airport, 54,250 SARS-CoV-2 tests were performed on arriving participants, of which 55 were positive (0.10%) by nasopharyngeal SARS-CoV-2 PCR and were sent to an isolated quarantine facility. Of the 1,014,170 screening tests conducted during the Olympic and Paralympic Games, 299 were confirmed positive (0.03%). Approximately 11,000 participants stayed in the Olympic Village during the Olympic Games and 4,400 participants stayed in the Olympic Village during the Paralympic Games; 3,426 SARS-CoV-2 PCR tests (0.34% of all screening tests) were performed on close contacts in the Olympic Village, and 15 results were confirmed positive (0.44%); 7 of 1,508 tests during the Olympic Games [ 0.46%] during the Olympic Games and 8 of 1,918 tests [0.42%] during the Paralympic Games). The number of SARS-CoV-2 PCR tests in close contacts increased after the opening of both the Olympic and Paralympic Games. During the Olympic Games, the number of SARS-CoV-2 PCR detections increased from 70 on the opening day (day 1), peaked at 142 on day 5, and then decreased. During the Paralympic Games, the number of SARS-CoV-2 PCR detections increased from 122 on day 1 to a peak of 166 on day 5, and then decreased to 33 on day 3.15 Positive cases were distributed between the Olympic and Paralympic Games, and there were no major clusters of cases. | All participants (e.g. athletes, coaches, medical staff, physiotherapists and Paralympic chaperones) are required to be tested negative for SARS-CoV-2 before leaving their country of origin. Vaccination against COVID-19 is recommended but not mandatory. All persons were tested for SARS-CoV-2 salivary antigen upon arrival at Japanese airports and were immediately transferred to the Paralympic Village if the results were negative. A number of public health measures were implemented to control the spread of SARS-CoV-2 in the Paralympic Village, including behavioral interventions such as wearing face masks.3 Almost all participants were required to stay in the Paralympic Village except during training and competition. All participants underwent laboratory saliva antigen screening each morning. Those who screened positive underwent confirmatory nasopharyngeal polymerase chain reaction (PCR) testing.Individuals who tested positive for PCR were moved to a quarantine hotel outside the Paralympic Village, where they were not allowed to have contact with others. |  |  |  |  |  |  |
| Actual situation of handling Tokyo 2020 Games-related patients at a designated hospital during COVID-19 pandemic |  |  |  |  | (18) COVID-19: The most common reason for consultation was new crown pneumonia (8 patients, 5 of whom were hospitalized). In SARS-CoV-2 screening, 55 cases of COVID-19 were diagnosed in 54,250 tests at airports for people coming to Japan from overseas to participate in the 2020 Tokyo Olympics, and 299 cases were diagnosed in 1,014,170 tests during the Olympics and Paralympics. |  |  |  |  |  |  |  |
| What has the 2020 Tokyo Olympic and Paralympic Games taught global health on sporting mass gatherings under COVID-19 pandemic? |  |  |  |  | As of September 8, 2021, a few days after the conclusion of the Paralympic Games, the Tokyo 2020 Organizing Committee officially reported 866 cases of COVID-19, with athletes, competition-related personnel, journalists, and contractors accounting for 41, 201, 53, and 502 cases, respectively, and more than two-thirds of the cases originating from Japanese residents. | 1. Regular COVID-19 screening (not yet widely available to the public in Japan), wearing masks, and increasing the vaccination rate of participants;  2. participants are asked to follow the mitigation measures specified in the IOC manual. In addition, national epidemiologic surveillance of infectious diseases has been strengthened since July 2021 to enhance early detection and response, and while there are few reports on the extent to which participants are following the PHSM guidelines, it is noteworthy that more than one million tests were conducted during the Games and that transmission of the virus among athletes and staff was relatively suppressed, in contrast to the COVID-19 outside of the Games venues This contrasts with the deterioration of COVID-19 outside the Olympic venues;  3. As of mid-July 2021, 85% of athletes, 100% of IOC officials, and 70-80% of media staff were reported to have been vaccinated, which helped to break the chain of transmission and mitigate risk among athletes and officials;  4. While vaccination status is not a requirement for participation in the Tokyo Games, it is expected that a vaccine passport combined with a negative COVID-19 test result and regular screening will be necessary, along with appropriate measures such as masking. These measures apply not only to athletes and officials, but also to spectators. In summary, the following takeaways are key lessons learned from Tokyo 202Q: Organizers of the Olympic and Paralympic Games should conduct rigorous health risk assessments prior to the Games through an all-hazards approach, in collaboration with global public health agencies and organizations. - At a minimum, host countries should contain the spread of viruses beforehand. - Regular screening facilitates the rapid interruption of the chain of transmission of the virus. - It is important to ensure emergency response capacity for the Games and access to testing and health care for citizens - this will facilitate appropriate risk communication and promote community engagement. - Ensuring accountability and transparency in risk analysis, data collection and reporting is essential to promote open scientific discussion, thus contributing to the safe management of the Games. |  |  |  |  |  |  |
| Athlete Medical Services at the Marathon and Race Walking Events During Tokyo 2020 Olympics | Data are first recorded on paper charts and then entered into an electronic system for statistical analysis. | (11) Heat-related illness: A total of 50 athletes were transferred to the Athlete Medical Station: 28 athletes finished the race (i.e., passed out after the finish line), while 22 athletes were DNF athletes transferred from the race site. Forty-eight (96%) of the athletes transported to the Athlete Medical Station exhibited signs and symptoms of EHI. HE was most common (n = 22), followed by EAMC (n = 12), HS (n = 12), and EHS (n = 2). (AMS, Athlete Medical Services; DNF, incomplete; EAMC, exercise-associated muscle cramps; HE, heat stroke; HS, heat syncope; CWI, cold water immersion.) |  |  |  | 1. Exertional Heat Stroke (EHI) Recognition and Treatment The Athlete Medical Station is equipped with rectal thermometers and ice baths to provide cold water immersion when a collapsed Athlete is diagnosed with Exertional Heat Stroke. After a collapsed athlete is admitted to the Athlete Medical Station, a medical volunteer trained in pre-hospital care for exertional heat stroke determines the need to assess the athlete's internal temperature using a rectal thermometer (DataTherm II Continuous Temperature Monitor, Geratherm Medical AG). Athletes diagnosed with hyperthermia (rectal temperature ≥40.5◦ C) and central nervous system dysfunction were cooled by whole-body cold water immersion. The water temperature in the ice bath was maintained between 10 and 15◦ C. The water temperature in the ice bath was maintained between 10 and 15◦C. Rectal temperature is monitored throughout the cooling process and stopped when the rectal temperature reaches <39◦ C. EHS diagnosis and treatment is performed by physicians, nurses, and physical therapists;  2. at a minimum, the contingency plan should address arrangements for event and medical volunteers, traffic and road closure control, spectator control and safety, ambulance service, and broadcast and media relations. |  |  |  |  |  |  |
| Non-SARS-CoV-2 Respiratory Viruses in Athletes at Major Winter Sport Events, 2021 and 2022 | Laboratory Testing. | (1) Certain infectious or parasitic diseases: The panel detects the following viruses: Respiratory syncytial virus; Adenovirus; Influenza A and B viruses; Rhinovirus/Enterovirus; Parainfluenza viruses 1-4; Human Coronavirus 229E, OC43, HKU1, and NL63; SARS-CoV-2; Middle East Respiratory Syndrome Coronavirus; and Human Subpneumovirus. We documented 6 symptomatic cases of ARI and found the virus in 4 of them. We detected 1 respiratory syncytial virus in 1 athlete on day 1, 1 subpneumovirus in 1 staff member on day 2, and 1 coronavirus 229E in 1 athlete on day 3. 1 staff member developed ARI after returning to Finland and was identified as coronavirus OC43 |  |  |  | OVID-19 Response measures include: relative isolation of team members prior to travel (i.e., wearing masks and maintaining body distance); negative SARS-CoV-2 testing prior to departure; wearing masks during travel and games; traveling on chartered flights; increased hand hygiene and environmental disinfection; maintaining body distance; staying in single or double rooms; limiting the use of indoor public facilities; and in Beijing Limit admission to a small number of spectators. All teams in Beijing have been vaccinated with COVID-19. |  |  |  |  |  |  |
| Incidence and factor analysis for the heat-related illness on the Tokyo 2020 Olympic and Paralympic Games | (1) Medical Operations Manager (MOM) daily reports (number of cases handled by each venue's assigned MOM), (2) J-SPEED (Survey of Extreme Conditions) data (Non-Athlete Response Reports) and (3) Electronic Medical Record (EMR) data (Athlete Medical Records). Other records included in the analysis included (4) destination hospital visit data, (5) Major Operations Center - Functional Coordination Center data (Medical Coordination Unit Coordination data) and (6) destination hospital insurance medical records from the Organizing Committee Medical Department and Sports Authority. | (11) Heat-related illnesses: In the Olympic Games, the incidence of heatstroke in the marathon/racewalking event (Odori Park, Sapporo), which is a high-intensity, prolonged exercise, was 4.48% (50 cases), and the incidence of heatstroke in the short-distance track and field event at the Olympic Stadium was 0.13% (20 cases), for a combined rate of 4.61% with the track and field event. The cycling and marathon swimming events followed with incidence rates of 2.78% and 1.96% respectively. In the Paralympics, the triathlon event had the highest incidence rate of 1.47%, while the track and field and marathon events had an incidence rate of 0.17%. |  |  |  | 1. Athletes, National Olympic Committees (NOCs), International Federations (IFs) and others took measures to protect themselves from the heat, including the provision of cooling undershirts for athletes and officials with disabilities, the preparation of athletes' lounges, portable coolers, and the installation of fans and cool-mist fans in various parts of the venues. For athletes in wheelchairs, coolers were placed on the mounting surfaces of their torsos. In addition, oral rehydration salts and other beverages were prepared at each venue to protect against heat-related illnesses, and tablets were distributed to encourage replenishment of essential salts. In the event of heat-related illnesses, athletes were quickly taken to the Clinic (the medical clinic at the competition site) for initial treatment. In particular, cooling with cooling towels, intravenous drips and oral rehydration salts were effective in replenishing extracellular fluids. In 12 high-intensity sports and 18 high-temperature, high-risk sports, medical ice baths are prepared in the medical rooms of the competition venues. High-temperature athletes who have been carefully trained to have a rectal temperature of 39.5°C or more are quickly immersed in cold water;  2. In the Olympic Games, there were only 8 cases (8%) in rowing and beach volleyball and 5 cases in the Paralympic Games, including triathlon, track and field, rowing and futsal, in addition to 6 cases in endurance sports (marathon, race walking), suggesting that prevention of severe heatstroke due to cooling may help to reduce the burden on local healthcare facilities during a COVID-19 pandemic;  3. Despite the fact that the competition was moved from Tokyo to Sapporo, where the WBGT was higher, and that the competition started early in the morning, 50 cases of heat-related illnesses occurred during the endurance event. However, sufficient precautions were taken to reduce the risk of athletes developing heat-related illnesses without causing serious illness. In tennis, matches were postponed, the women's soccer final was changed from daytime to nighttime, and the venue was changed. In golf, matches were moved up or shortened;  4. Thorough heat stroke precautions and early cooling of athletes resulted in the avoidance of emergency medical care during this period. It cannot be denied that heat stroke would have been a major risk if spectators had been present. |  |  |  |  |  |  |
| Heading' in the right direction: concussions reported at the Tokyo 2020 Paralympic Games | The Web-based Injury and Illness Surveillance System (WEB-IISS) used by team medical staff during the Paralympics has been developed and updated over time to cover clinically relevant issues. To enhance concussion reporting, the WEB-IISS has included concussion-related information for all head/face and neck injuries reported by the system. For athletes without team medical support, concussions will be reported through the playing field medical staff or through the medical services of the Comprehensive Clinic. |  |  |  |  |  | Nine concussions were reported during the Tokyo Olympics (eight through WEB-IISS; one through a polyclinic). These concussions accounted for 6 of the 16 head and face injuries and 3 of the 13 neck injuries. |  |  |  |  |  |
| Effects of a closed-loop system against SARS-CoV-2 at the Beijing 2022 Olympic Winter Games: a descriptive and modeling study |  |  |  |  | Between January 1, 2022 and February 20, 2022, 280 SARS-CoV-2-positive participants transported by Beijing EMS were enrolled in this study. Ninety-three (33.2%) cases were detected by initial screening, and the remaining cases were detected by routine testing in a closed-loop system. The diagnostic interval of all cases was reviewed and 274 (97.9%) cases were found to be imported cases (i.e., detected within the first 14 days of the closed-loop system), whereas only 6 (2.1%) cases were infected in the circulatory system. | Vaccination, restriction of social interactions, PCR testing, masking, and isolation of confirmed cases have been effective in stopping the spread of neo-coronaviruses within closed-loop systems. |  |  |  |  |  |  |
| The Impact of Preventive Strategies Adopted during Large Events on the COVID-19 Pandemic: A Case Study of the Tokyo Olympics to Provide Guidance for The Impact of Preventive Strategies Adopted during Large Events on the COVID-19 Pandemic |  |  |  |  | The postponed Tokyo Olympics were successfully held from July 23 to August 8, 2021 in the face of these foreseeable challenges. Only 41 athletes and 822 non-athletes tested positive for COVID during the Tokyo Olympic and Paralympic Games. | 1. frequent testing programs and bubble programs; no spectators are allowed to attend the Games except in some remote areas outside Tokyo; COVID-19 vaccination is encouraged, but not mandatory, for all Games participants;  2. use of competition-specific transportation; official accommodations; daily temperature monitoring for 14 days; Various infection risk models have been developed to quantitatively assess the level of risk of infectious disease transmission, such as the susceptible-infected-recovered (SIR) model, the environmental exposure model, and the logistic model. |  |  |  |  |  |  |
| [Application of mathematical models of infectious diseases in the evaluation of COVID-19 transmission risk at mass gatherings] |  |  |  |  | The number of cases involving Austria in China was 323, and the actual number of cases detected in the closed loop was 212. |  |  |  |  |  |  |  |
| Team USA COVID-19 Prevalence at Tokyo 2020 Summer Olympic and Paralympic Games | Laboratory monitoring |  |  |  | Between 1% of Team USA Olympic and Paralympic athletes and staff were diagnosed with COVID-19 and quarantined, and between 1% and 10% were quarantined after being determined to have had close contact with an individual diagnosed with COVID-19. Olympic surveillance: 8 athletes, 10 staff infected; Paralympic: 23 athletes, 7 staff infected. | Tokyo 2020 COVID-19 Mitigation Measures The IOC and TOC established a Joint Steering Committee and developed an Action Manual to guide athletes and National Olympic and Paralympic Organizations in developing policies and procedures to mitigate the spread of COVID-19 during the Games. The Action Manual focuses on four principles: (1) masking; (2) minimizing physical contact; (3) detection, tracing and isolation; and (4) hygiene practices. In addition, a "bubble system" was implemented to separate athletes, support staff and TOC volunteers from the general public, and strict infection prevention measures outlined in the Action Manual were implemented. Spectators were largely barred from entering the Games and the total number of spectators at the Games was limited to approximately 43,300, despite the significant economic cost to Japan. |  |  |  |  |  |  |
| Evaluation of effectiveness of COVID-19 prevention and control in a closed-loop management manner during Beijing 2022 Winter Olympics and Paralympics Games |  |  |  |  | During the Tokyo 2020 Olympic Games, out of more than 600,000 nucleic acid tests conducted, there were a total of 436 cases of new coronavirus pneumonia among people involved in the games, which is much lower than the rate of new coronavirus infections in Japan during the same period. Airport arrivals and nucleic acid tests. As of March 13, a total of 7,777 athletes and officials accompanying their teams and 10,901 other stakeholders, for a total of 18,678 persons, had entered the airport. From January 27 to February 3 and from February 25 to March 3 (from the opening of the Winter Olympic Village and the Winter Paralympic Village to the Opening Ceremony, respectively), the number of daily arrivals reached a peak, with 10,503 (56.23% of all arrivals) and 2,081 (11.14%), respectively. 323 people tested positive from January 4 to March 13, of which 131 were infected by athletes and officials accompanying their teams, and 131 were infected by other stakeholders. persons and 192 persons infected by other stakeholders. The cumulative detection rate of incoming persons was 1.73%, of which 1.68% and 1.76% were athletes and officials accompanying teams and other stakeholders respectively. As of March 13, 150,990 detections were made by athletes and officials accompanying teams and 2,737,528 by other parties of interest in the closed loop, totaling 2,888,518 detections. During this period, there were a cumulative total of 212 positive tests for the new coronavirus, of which 74 were positive for Athletes and Team Officials and 138 were positive for Other Stakeholders. |  |  |  |  |  |  |  |
| Completion rates and injuries in alpine races during the 1994 Olympic Winter Games |  |  |  |  |  |  |  |  | Injuries have included a concussion in an obstacle course race, a talus fracture in a superb obstacle course race, and a ruptured anterior cruciate ligament (ACL) in the knee during a downhill training race. |  |  |  |
| The epidemiology of illness and injury at the alpine venues during the Salt Lake City 2002 Winter Olympic Games | Intermountain Health Care, known for its expertise in medical informatics and a long tradition of computerized medical records, developed, populated, and maintained the database of Olympic medical services used during the Games. At the time of the evaluation, a "medical visit form" was generated for each patient visit to any venue clinic. | (7) Respiratory diseases: 88 cases in the respiratory system. | (6) Diseases of the digestive system: 52 cases of the digestive system. | (16) Multiple systems: diseases of the ear, nose and throat (n 29). |  | The Salt Lake City Olympic Committee mitigated the effects of the cold on spectators by conducting an aggressive public awareness campaign and frequent radio and television broadcasts of weather conditions to encourage the use of winter gear. |  |  | Sprains and strains (n 108), other trauma (n 103) Similarly, "miscellaneous trauma" was used to indicate other types and locations of injuries. These include: lacerations (n 27); contusions or abrasions (n 37); thoracolumbar spine injuries (n 10); cervical spine injuries (n 3); burns (n 5); closed head injuries (n 5); and other (n 16). |  |  |  |
| Nordic venue medical services during the 2002 Winter Olympics | Medical evaluations and treatments provided by physicians or mid-level practitioners during the 2002 Winter Olympics were documented on medical intake forms. Medical intake forms were completed for all patients treated in the Athletes' Clinic and some patients treated in the Spectators' Clinic. | (7) Respiratory diseases: Of all the patients treated at the Athletes' Medical Clinic, the most common diagnosis was "febrile respiratory infection" (17 patients), followed by "other respiratory diagnoses" (11 patients). In the spectator medical clinic, the most common diagnosis was also "feverless respiratory infection" (31 patients). | (11) Heat-related illnesses: There were three cases of cold-induced limb injuries (frostbite), all of which were minor. |  |  |  |  |  |  |  |  |  |
| Injuries in team sport tournaments during the 2004 Olympic games | The injury reporting system used in this study was developed to record injuries and illnesses during team sport tournaments and has been implemented as a routine practice in more than 20 international soccer and handball tournaments. The injury report form consists of a single page, which describes in tabular form all injuries that occurred or did not occur (if applicable) during a given tournament. |  |  |  |  |  |  |  | Almost half of the injuries (n = 171, 46%) were diagnosed as contusions. Sprains (n = 48, 13%) and strained muscle fiber ruptures (n = 35, 10%) were also common. 17 injuries were diagnosed as fractures; 17 were diagnosed as ligament ruptures; 8 were diagnosed as dislocations; 7 were diagnosed as concussions; and 4 were diagnosed as meniscus injuries. |  | Overall, males and females sustained similar injury sites and conditions. However, there was a significant difference in the types of injuries between the two groups (P < .05), with female players sustaining more concussions (4% vs 0.5%) and sprains (19% vs 9%) and fewer fractures (3% vs 6%) and lacerations (5% vs 11%) than male players. |  |
| Wrestling injuries during the 2008 beijing olympic games | Use a standardized checklist to collect injury data, including type of injury, severity, location, time and mechanism. |  |  |  |  |  | (1) Injury incidence rate: equates to a total incidence of 9.30 injuries per 100 athletes and 7.88 injuries per 100 games. |  | Recorded injuries consisted mainly of 19 skin lacerations (59.4%), 4 nose bleeds (12.5%), 8 sprains (25%) and 1 muscle strain (3.1%). |  |  |  |
| Injuries in judo: A systematic literature review including suggestions for prevention | Looking for prospective and retrospective studies on judo sports injuries. |  |  |  |  |  | The most common injuries were sprains (5.6-59.8%), strains (7-33.8%) and contusions (5.6-56%; see Online Supplementary Tables S2 and S3). Judo injuries mainly affected the body extremities, especially the knees (up to 28%), shoulders (up to 22%) and fingers of the hands (up to 30%), as shown in Online Supplementary Table S4. The risk of time-loss injuries in judo during the Olympic Games is not high: in the last two Olympic Games, the average risk of injury was 6.4-8.9%. | The percentage of sprains was slightly higher in female judokas and strains in male judokas. | The most common injuries were sprains (5.6-59.8%), strains (7-33.8%) and contusions (5.6-56%). |  |  |  |
| The Epidemiology Of Injuries In Paralympic Athletics (Track And Field) At The London 2012 Paralympic Games | Daily injury data were obtained through 2 databases: (1) a customized Web-based Injury and Illness Surveillance System (WEB-IISS), which is maintained by team medical staff; and (2) the Organizing Committee database, which is maintained by medical providers at medical stations operated by the London Organizing Committee for the Olympic and Paralympic Games. Athlete injuries and event discipline were obtained through the International Paralympic Committee athlete database. |  |  |  |  |  | Athletics had an IR of 22.1 for the entire period |  | The highest percentage of thigh injuries in ambulatory athletes (16.4% of all injuries; IR, 4.0) occurred primarily in track and field athletes. The highest percentage of shoulder/clavicle injuries (19.3% of all injuries; IR, 3.4) occurred in wheelchair-bound or seated athletes, primarily in field athletes. |  |  |  |
| The Epidemiology of Injuries in Football at the London 2012 Paralympic Games | The Paralympic Injury Surveillance System is used to track injuries and illnesses during the Games, with data entered by medical staff. |  |  |  |  |  | Futsal: 22 injuries were recorded during the 14 days of the Olympic Games, resulting in an IP of 31.4 injuries per 100 athletes) and an IR of 22.4 injuries per 1000 athlete-days.  Soccer Sevens: 14 injuries were recorded during this period, resulting in an IP of 14.6 injuries per 100 athletes and an IR of 10.4 injuries per 1000 athlete-days. |  |  |  | Futsal: The lower extremities were the most common site of injury. The head and neck accounted for 25% of acute competition injuries and 18% of all injuries, with the knee being the most common site of injury (Table 2).  Soccer sevens: 12 of the 14 injuries (86% of all injuries) involved the lower limbs. The ankle (36% of all injuries) and knee (29% of all injuries) were the most commonly injured parts of the body. Five injuries (36% of all injuries) were due to contact with other players. |  |
| The epidemiology of injuries in powerlifting at the London 2012 Paralympic Games: an analysis of 1411 athlete-days | This was done by sub-analysis of the Integrated Injury and Illness Surveillance Study (IIIS) conducted during the London 2012 Paralympic Games. A more complete description of the methodology used for the Integrated Injury and Illness Surveillance Study has been described in detail elsewhere. |  |  |  |  |  |  | There were no significant differences between male and female weightlifters. The highest IR was found in the 26-34 years age group followed by the youngest age group (13-25 years) compared to the oldest age group (35-67 years) but these differences were not statistically significant. |  |  | The most common site of injury was the shoulder clavicle (31.6% of all injuries), followed by the elbow and chest (13.2% of all injuries each), upper arm and ankle (7.9% of all injuries each), and cervical spine (5.3%). |  |
| Mitigating risk of injury in alpine skiing in the Pyeongchang 2018 Paralympic Winter Games: The time is now! |  |  |  |  |  |  |  |  |  |  |  |  |
| Gymnastics injury incidence during the 2008, 2012 and 2016 Olympic Games: Analysis of prospectively collected surveillance data from 963 registered gymnasts during Olympic Games | The NOC Chief Physician and the Olympic Organizing Committee Medical Team report the occurrence of new injuries on a daily basis on a standardized reporting form. |  |  |  |  |  |  | More injuries occurred during training (49.4%) compared to competition (39.5%) (11.1% missing information). | The most common type of injury was sprains (34.6%), followed by tendinopathy/arthritis/impacts (17.3%), contusions (9.9%), and fractures (7.4%). |  | In this study, ankle and ligaments were the most common sites of injury and ankle was the most common site of injury followed by lumbar spine, foot, hip and groin. |  |
| A systematic review of injuries in gymnastics | We searched seven electronic literature databases and two gray literature databases. Two reviewers independently assessed titles/abstracts, extracted data. |  |  |  |  |  |  | 49% of the injuries occurred during training and 40% occurred during competition (missing information 11%). | The type of injury was sprains (35%), followed by tendinopathy/arthritis/impact (17%), contusions (10%) and fractures (7%). | Mackie reported in 1994 that (1) Canadian national team gymnasts have a higher incidence rate than provincial gymnasts and hypothesized that high level gymnasts are at a higher risk of injury per unit of training time due to the technical difficulty of the movements; (2) injuries predominate in the sport of freestyle gymnastics because athletes spend most of their time on the floor and during warm-ups; (3) the balance beam exercise are becoming more complex, leading to an increase in injuries; (4) injuries after falls and dismounts are caused by gaps between the landing mat and the mat, which is too small; (5) young gymnasts often suffer from overuse injuries over a long period of time, which should be prevented by performing Achilles tendon, hamstring, and quadriceps stretching exercises; and (6) avoiding hyperextension on the landing can prevent intervertebral disc stress fractures. | Injuries were predominantly to the lower limbs in 63% of cases, followed by the trunk in 23% and the upper limbs in 14%. |  |
| Injury Epidemiology and Preparedness in Para Powerlifters at the Rio 2016 Paralympic Games: an Analysis of 1410 Athlete-Days | This cohort study is a sub-analysis of the Web-based Injury and Illness Surveillance System Survey (WEB-IISS) conducted by the International Paralympic Committee (IPC) Medical Committee. |  |  |  |  |  |  |  |  | 1. age is a significant risk factor for injury at the Rio Olympics;  2. the use of the shoulder as a weight-bearing joint by athletes, coupled with the unique athletic demands of the bench press, may explain these findings. Individuals who use manual wheelchairs, canes, and/or crutches on a daily basis place high loads on the shoulder joint due to the frequent repetition of daily tasks including center of gravity transfers, decompression lifts, transfers, propulsion on flat and inclined surfaces, and start/stop maneuvers. Although both seated and walking athletes exert stress on the shoulder during ADLs (e.g., grooming and dressing), the shoulders of seated subjects are subjected to much higher stresses due to their simultaneous role as weight-bearing joints. Unusually high forces during activities of daily living contribute to common repetitive motion injuries in disabled weightlifters;  3. the heaviest male athletes who lift the heaviest weights have the highest injury rates, which supports the hypothesis that repetitively applying very high loads puts the musculoskeletal system at higher risk for overload injuries. Finally, in Rio, advancing age was a significant risk factor for injury. | shoulders |  |
| Injury incidence, severity and profile in Olympic combat sports: a comparative analysis of 7712 athlete exposures from three consecutive Olympic Games |  |  |  |  |  |  | A total of 315 competition injuries occurred among combat sports athletes during the three Olympic Games. A total of 315 match injuries occurred among combat sports athletes during the three Olympic Games. | Compared to males, female taekwondo athletes had a lower IIRME (RRME 0.69), whereas female judo athletes had a higher IIRME (RRME 1.35); however, none of the differences were statistically significant in either sport. Compared to lightweight athletes, heavyweight judo athletes had a significantly lower IIRME (RRME 0.57), while heavyweight boxers had a higher IIRME (RRME 1.32), although the difference was not significant in boxing. | The most common types of lesions were joint sprains (31.4%), lacerations and abrasions (23.2%) and contusions (19.7%). | 1. The IIRME for male boxers tripled (RRME 3.00) after rule changes were introduced after the 2012 London Olympics (i.e., elimination of protective head guards);  2. differences in injury profiles across combat sports are attributed to the unique characteristics of these sports, such as the rules of the game (e.g., permitted techniques and targets, mandatory protective equipment). For example, while both boxing and kickboxing allow strikes to the head and torso, only the latter allows the use of kicks. This increases the typical distance between opponents and reduces the frequency of strikes, resulting in fewer head impacts and more distal lower extremity injuries in taekwondo compared with boxing. | Overall, the most common site of injury was the head and neck (35.9%), followed by the upper extremities (31.1%) and lower extremities (26.3%). The highest and lowest proportions of head and neck injuries were in boxing (62.1%) and taekwondo (4.8%). Upper extremity injuries were particularly common in judo (42.6%), while the lower extremity was the most common site of injury in taekwondo (59.5%) and wrestling (45.5%). |  |
| Medical care provision at the venue of the weightlifting event of the Tokyo 2020 Olympic Games | The weightlifting competition was held from July 19 to August 4, 2021 at the TIF. A notebook was prepared for each station assigned to a paramedic, and each station recorded the date and the gender and country of the patient. In addition, the location and treatment details were recorded at the first aid station. At the medical station or FOP, the patient's age, chief complaint, and whether or not the patient was referred to a support organization were recorded. |  |  |  | (18) COVID-19 (SARS-CoV-2): A total of 194 athletes participated in the Olympic weightlifting competition, excluding three athletes who were unable to compete due to injury or SARS-CoV-2 infection. | 1. Previous reports have shown that all athletes have had at least one illness and poor mental health prior to the Olympic Games. It should therefore be noted that more athletes are at risk, both physically and mentally, than would be expected;  2. analysis of athletes competing in the Olympic Games reported that being female, low energy availability and poor mental health were risk factors for injury. It is therefore vital to have a full understanding of the medical examinations that athletes receive;  3. sometimes inexperienced staff are unable to treat well. It is therefore important to know in advance what measures need to be taken at each competition venue. |  |  |  |  | Knees, fingers and wrists are the most commonly treated areas at the training ground first aid station, while fingers, knees and calves are the most commonly treated areas at the first aid station in the warm-up area. |  |
| Activities of medical centers for athletes and spectators at cycling track events in the Tokyo 2020 Olympic and Paralympic Games | Two medical clinics were set up for the track cycling competition: one for Olympic athletes and their colleagues, and one for spectators, TOC-related personnel and volunteers. |  |  |  |  |  |  | Six patients were diagnosed as follows: facial contusion, n = 1; rib fracture, n = 1; clavicle fracture, n = 1; distal radius fracture, n = 1; and scapula fracture, n = 1. |  |  |  |  |
| Medical Care Management Based on Disaster Medicine for the Triathlon Events at the XXXII Olympiad and Tokyo 2020 Paralympic Games |  |  |  |  |  | 1. The two components of the Tokyo Olympic Games Plan were (1) raising awareness of high temperatures and providing information such as wet bulb black bulb temperatures at major venues in multiple languages; and (2) working with the Organizing Committee and the Tokyo Metropolitan Government to maximize heat stroke measures during the Games. In addition, the plan mentions the compatibility of dual measures against epidemics and heatstroke; ice baths, drinking water, and ice for cooling were prepared in large quantities and made available at more stations than usual. Staff were required to wear full personal protective equipment (PPE) to prevent infection in extreme conditions, which included hats, masks, face masks, gloves and gowns. As a result, shifts were created to help staff take more frequent breaks than usual, and they were instructed to drink water regularly;  2.Chad A. Asplund et al. advocate that under normal circumstances, the medical director should play the following roles in a triathlon medical assistance program:(1) communicate with organizers to cover all aspects of safety, such as course safety; (2) develop clinical protocols; (3) recruit and train medical teams at the goal and on the course; (4) organize the medical facility, including providing supplies for it; and (5) prepare for the athletes receiving medical care; (6) organizing documentation protocols; and (6) advocating for communication and coordination with EMS and local hospitals;  3. As part of the overall infection control measures for the Tokyo 2020 Olympic Games, even athletes will be required to wear masks inside the venues when not competing and will be required to undergo a PCR test every time they enter or leave a venue from outside the bubble. The organizing committee received daily activity reports from infected persons, which allowed for the identification of those in close contact and attention to their behavior. The venue is also well stocked with hand sanitizers. In addition, face shields, masks and gloves were standard equipment for OMP medical staff and protective clothing was worn during CPR and other centralized contact procedures. In addition, medical personnel are required to report physical status before and after work each day. |  |  |  |  |  |  |
| Sports dentistry and the olympic games |  | (15) Dentistry: Dentistry was the fourth most popular service at the Polyclinic during the 1998 Nagano Winter Olympics, accounting for nearly 20% of all visits. A total of 260 dental patients received 164 restorations, 35 root canals and 7 extractions. 17% of the patients were from Russia and Ukraine and 9% were from Russia and Ukraine. Seventeen percent of the patients were from Russia and Ukraine, and another 9 percent were from Kazakhstan. |  |  |  | 1. Dental treatment modalities: Since dentists are volunteers in most cases, there is little ancillary cost other than food and equipment. With more staff, chairs and time, more teeth can be saved and fewer extractions can be done; the most effective and efficient delivery system is to set up proper dental clinics within the village polyclinics. This ensures convenience and safety and allows the use of shared services such as reception and interpreters. Joint consultations with other healthcare colleagues on difficult cases can be held at any time. The clinic can look like a general dental clinic, allowing athletes to feel it is more specialized;  2. Physical set-up of the dental clinic: The ideal dental clinic should have a reception area, an appropriate number of operating rooms, an area for cleaning and sterilizing instruments, a storage area and a small laboratory area. It should be equipped with the latest equipment and supplies, digital apical and panoramic X-rays, air abrasion, laser technology, electronic apical positioning and other state-of-the-art equipment, as well as all available modern restorative materials, bonding techniques and cements;  3. Services provided: The most common services are examinations, X-rays (both apical and panoramic), restorations (permanent and temporary), root canals (complete or partial), hygienic or periodontal treatment and extractions. The practice must also be prepared to deal with all potential trauma, including reimplantation of lost teeth and immobilization of dislocated teeth. Once stabilized, severe trauma or fracture of the alveolar bone can be referred to an outside clinic or hospital;  4. Dental Protection Programs: With major advances in dental protection technology and evidence of their effectiveness, many organizations now officially recognize or mandate the use of these protective devices. At the Summer Olympic Games, athletes competing in basketball, field hockey, boxing, martial arts, volleyball and soccer should have access to a mouthguard program. At the Olympic Winter Games, Athletes competing in ice hockey, steeplechase, aerials and snowmobiles should be provided with pressure-laminated mouthguards;  5. Competition Venue Coverage: Dental clinics located far from the competition venues are not conducive to the rapid return of Athletes to the field of play, particularly in light of current trauma protocols that require reimplantation within five minutes for optimal success. These dental emergencies should be treated at the competition site by experienced and well-equipped sports dentists. In Olympic sports, consideration should be given to having on-site dentists in hockey, boxing, and some martial arts sports (e.g., taekwondo). On-call or on-site dentists may also be required for basketball, field hockey and soccer (especially in medal competitions);  7. staffing: clinic staff should include general dentists, hygienists, dental assistants, and specialists in endodontics and oral surgery.  8. other recommendations: many authors, including the British Dental Association, have reported on the potentially harmful effects of sports drinks on athletes' teeth. This damage can be reduced or eliminated if athletes also have access to water. |  |  |  |  |  |  |
| Dental data of the athens 2004 olympic and paralympic games |  | (15) During the Dental Olympics, more than 1,400 dental cases were treated by more than 650 patients, elite athletes, escorts, coaches and Olympic Village staff. Of these, 313 fillings, 100 root canals, 57 mouth guards and 9 dental trauma cases were treated. During the Paralympic Games, more than 240 dental cases were treated on more than 220 patients. Of these, 73 fillings, 12 root canals, 21 extractions and 3 traumas were treated. |  |  |  |  |  |  |  |  |  |  |
| Foot and ankle injuries during the Athens 2004 Olympic Games |  |  |  |  |  |  |  | The highs and lows of match injuries in men and women were more in patients 358 (58%) males than 266 (42%) females. 84.1% of the consulted cases were related to musculoskeletal injuries. | Tendonitis was the most common reason for visits, followed by ankle sprains, infected nail injuries, little toe sprains and stress fractures. There were significant differences in the types of injuries sustained in different team sports, with a higher incidence of serious injuries such as fractures and ligament injuries in soccer, basketball, handball, steeplechase and volleyball compared to other sports. |  |  |  |
| Dental ser vice in 2008 summer olympic games | 1. reception card (RCd); 2. medical history file (PF); 3. medical consultation form (Med-E) provided by the IOC. | (15) Dentistry: The most common procedures in dental care for the Beijing Olympics were permanent fillings, root canals, hygiene, treatment of pericoronitis in blocked teeth, and provision of new retainers.155 participants received dental examinations and oral health education. There were 47 cases of extractions. Soft tissue trauma (four cases) and dental trauma (two cases) were treated. Two athletes were found to have temporomandibular joint disorder (TMD) and were treated. Six patients were treated for abscesses on an emergency basis. Three patients were found to be missing orthodontic clips. |  |  |  |  |  |  |  |  |  |  |
| Seeing you through London 2012: Eye care at the Olympics |  | (14) Ophthalmology: minor eye injuries, glaucoma, diabetic retinopathy and macular degeneration are the diseases detected and treated. Most patients came to the clinic for refractive status checks: 973 spectacles and 50 contact lenses were dispensed. |  |  |  |  |  |  |  |  |  |  |
| Epidemiologic Study of Shoulder Injuries in the PyeongChang 2018 Winter Olympic Games | During the Olympics, all medical records from venue medical centers and polyclinics were stored through electronic medical records (EMRs). We collected Olympic EMR data and patient information obtained from the Olympic medical services team about athletes complaining of shoulder pain. Basic information was collected for each patient, such as athlete registration number, age, nationality, date of birth, sport/event, date of visit, time/place and medium of injury, and competition/training. Medical information such as site of injury, mechanism of injury, diagnosis and laboratory findings were also collected. |  |  |  |  |  |  | A total of 14 athletes presented to the clinic with shoulder-related symptoms (9% of the total number of athlete injuries). Of these, 11 were male and 3 were female, with an average age of 24.5 years (17-32 years). 5 athletes were injured during a match and 9 were injured during training. | The most common diagnosis was contusion (n = 6), followed by rotator cuff injury (n = 3), superior labral anterior-to-posterior (SLAP) injury (n = 1), sprain (n = 1), acromioclavicular-rostral clavicle (AC-CC) injury (n = 1), dislocation (n = 1), and fracture (n = 1). |  |  |  |
| Eye clinic attendance at the olympic and paralympic games Rio 2016 and its correlation to the WHO indicators on eye health | Data were collected on the nationality of athletes attending eye clinics. | Reports of ocular findings during the Rio 2016 Olympic and Paralympic Games showed that refractive errors were the most common clinical finding, followed by cataracts. |  |  |  |  |  |  |  |  |  |  |
| Global Forum: Orthopaedic Physicians in the Winter and Summer Olympic Games |  |  |  |  |  |  |  |  |  |  |  |  |
| Incidence and burden of injury at the Tokyo 2020 Paralympic Games held during the COVID-19 pandemic: a prospective cohort study of 66 045 athlete days | Injury data were obtained daily through the established web-based injury surveillance system (WEB-IISS; 81 countries, 3836 athletes) and local organizing committee medical facilities (81 countries, 567 athletes). |  |  |  |  |  | 5-a-side soccer (17.2), taekwondo (16.0), judo (11.6), badminton (9.6). | Sixty-two athletes suffered from visual impairments (IP=22.5), 58 athletes suffered from physical impairments (IP=21.1), and 56 athletes suffered from spinal cord related disorders (IP=20.4). | The shoulder (n=46) was the most injured area, followed by the hand and fingers (n=40), knee (n=31) and lower leg (n=30). |  |  |  |
